# Native chemical ligation approach to sensitively probe tissue acyl-CoA pools

**DOI:** 10.1016/j.chembiol.2022.04.005

**Published:** 2022-07-21

**Authors:** Andrew M. James, Abigail A.I. Norman, Jack W. Houghton, Hiran A. Prag, Angela Logan, Robin Antrobus, Richard C. Hartley, Michael P. Murphy

**Affiliations:** 1 Medical Research Council Mitochondrial Biology Unit, University of Cambridge, Cambridge CB2 0XY, UK; 2 School of Chemistry, University of Glasgow, Glasgow G12 8QQ, Scotland, UK; 3 Cambridge Institute of Medical Research, University of Cambridge, Cambridge CB2 0XY, UK

**Keywords:** acylation, acyl-CoA, coenzyme A, cysteine, native chemical ligation, thioester, thiol, triphenylphosphonium

## Abstract

During metabolism, carboxylic acids are often activated by conjugation to the thiol of coenzyme A (CoA). The resulting acyl-CoAs comprise a group of ∼100 thioester-containing metabolites that could modify protein behavior through non-enzymatic *N*-acylation of lysine residues. However, the importance of many potential acyl modifications remains unclear because antibody-based methods to detect them are unavailable and the *in vivo* concentrations of their respective acyl-CoAs are poorly characterized. Here, we develop cysteine-triphenylphosphonium (CysTPP), a mass spectrometry probe that uses “native chemical ligation” to sensitively detect the major acyl-CoAs present *in vivo* through irreversible modification of its amine via a thioester intermediate. Using CysTPP, we show that longer-chain (C13–C22) acyl-CoAs often constitute ∼60% of the acyl-CoA pool in rat tissues. These hydrophobic longer-chain fatty acyl-CoAs have the potential to non-enzymatically modify protein residues.

## Highlights

• LC-MS/MS probe to detect the acyl moiety of thioesters using native chemical ligation
• Development of a quantitative and sensitive assay for ∼60 acyl-Co-As *in vivo*
• Longer-chain (C13–C22) acyl-Co-As often represent ∼60% of tissue acyl-CoA pools
• These longer-chain acyl-Co-As can modify protein *in vitro*

## Graphical abstract

**Figure:**
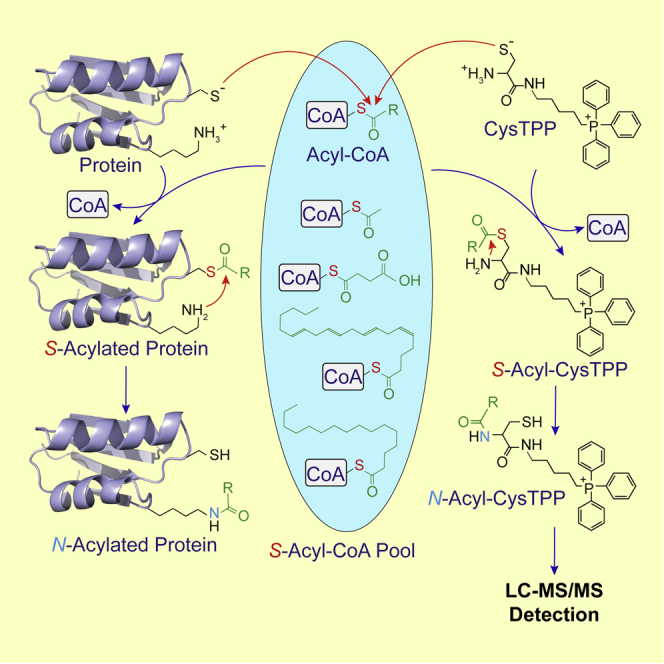

## Teaser

Acyl-Co-As can modify protein through non-enzymatic acylation. By using a novel mass spectrometry probe, James et al. demonstrate that longer-chain (C13–C22) acyl-Co-As often constitute ∼60% of the acyl-CoA pool in tissues.

## Introduction

Coenzyme A (CoA) is a key cofactor in many branches of metabolism. It contains a thiol that can condense with a carboxylic acid and form a range of activated acyl-CoAs. These reactive acyl-CoAs are metabolic intermediates in the oxidation of carbohydrate and fat in the mitochondrial matrix, as well as providing the building blocks for fatty acid and lipid synthesis in the cytosol (Pietrocola et al., 2015; Yu et al., 2018). However, acyl-CoAs can also *S-*acylate the thiol of cysteine residues and *N*-acylate the ε-amino of lysine residues, thereby affecting protein behavior. The importance of *N*-acylation is implied by the existence of several sirtuins (Sirt1–7), which remove acyl groups from protein lysines and are relevant to the pathology of a wide range of degenerative diseases, including cancer, aging, and diabetes (McDonnell et al., 2015; Pan and Finkel, 2017; Tabula Muris, 2020). *N-*Acetylation was initially considered solely as a regulatory modification that allows the cell to respond to acetyl-CoA, the acetyl-CoA/CoA ratio, or NAD^+^. However, the observation of several thousand sites of lysine *N-*acetylation *in vivo* with proteomics (Rardin et al., 2013; Weinert et al., 2015; Baeza et al., 2016), coupled with the vast majority having a very low (∼0.1%) stoichiometry of acetylation (Weinert et al., 2015, 2017; James et al., 2017; Hansen et al., 2019), suggests that regulation may be the exception rather than the rule (Prus et al., 2019). In addition to acetyl-CoA, several other acyl-CoAs (e.g., succinyl-CoA, malonyl-CoA, glutaryl-CoA) have been shown to generate *N-*linked modifications on lysines *in vivo* (Weinert et al., 2013; Peng et al., 2011; Tan et al., 2014). As few acyltransferases have been identified, it now seems likely that *N-*acylation at many of the thousands of sites observed with proteomics occurs non-enzymatically when the protonated amine group (p*K*_a_ ∼10.5) of a protein lysine deprotonates to become a nucleophile, and this attacks the thioester carbonyl of an acyl-CoA to generate a stable amide-linked modification (Wagner and Payne, 2013).

**Figure 1:**
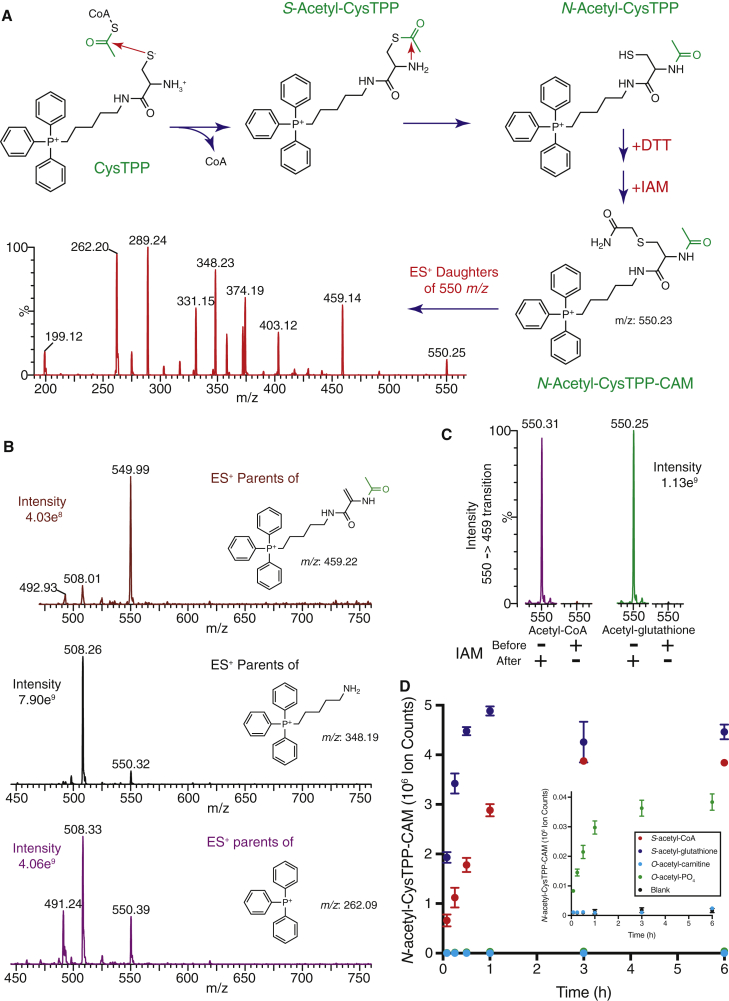
CysTPP detects acetyl-CoA (A) The CysTPP assay. Addition of acetyl-CoA to CysTPP results in a relatively rapid thioester exchange reaction generating CoA and an *S*-acetyl-CysTPP intermediate. The thioester carbonyl is then attacked by the proximal amine of CysTPP to generate *N*-acetyl-CysTPP. The high effective molarity of the amine greatly enhances what would normally be a slow S→N intermolecular reaction in bulk solvent (Kirby, 1980). Remaining *S*-acetyl-CysTPP is removed with dithiothreitol (DTT) and the free thiol is alkylated with iodoacetamide (IAM). The product at 550 *m/z* had a fragmentation pattern consistent with *N-*acetyl-CysTPP-CAM by MS/MS (see also Figure S2). (B) The 550 *m/z N-*acetyl-CysTPP-CAM product can be quantified using daughter ions at 262, 348, and 459 *m/z*. Parental peaks at 508 *m/z* and 491 *m/z* arise from non-acylated CysTPP-CAM and its δ-lactam, respectively. (C) Generation of *N-*acetyl-CysTPP-CAM proceeds via an *S-*acetyl-CysTPP intermediate. Alkylation of the thiol of CysTPP by preincubation with excess IAM prevents the formation of *N-*acetyl-CysTPP-CAM. (D) CysTPP reacts with thioesters, but not esters. *S*-acetyl-CoA and *S*-acetyl-glutathione transfer their acetyl moiety to CysTPP. In contrast, *O-*acetyl-carnitine and *O-*acetyl-phosphate do not react or react slowly with CysTPP. Data are the mean ± SEM (n = 3). See also Figures S1 and S2.

These non-enzymatic reactions have been suggested to represent a “carbon stress,” whereby slow but unavoidable non-enzymatic reactions on the surface of proteins may contribute to protein instability and aggregation (Wagner and Hirschey, 2014; Trub and Hirschey, 2018; Weinert et al., 2017; James et al., 2018b). This hypothesis is attractive as such non-enzymatic *N-*acylation of lysine residues could explain the benefits of sirtuins in degenerative diseases and aging (Kanfi et al., 2012; Satoh et al., 2013; McDonnell et al., 2015; James et al., 2018a). Supporting this interpretation, protein sites where a surface cysteine makes a nearby lysine susceptible to *N-*acylation are significantly less conserved within vertebrate genomes, suggesting that most *N-*acylation is detrimental (James et al., 2018a). While these surface sites of reactivity were identified using a dataset of lysine acetylation from mouse livers (Weinert et al., 2015), lysine acylation by any of the ∼100 acyl-CoAs present in mammalian metabolism could contribute to the observed detrimental impact (James et al., 2018b). However, the relative composition of the acyl-CoA pool in aggregation-prone tissues, such as brain tissue, is not well characterized (Deutsch et al., 1994; Blachnio-Zabielska et al., 2011; Palladino et al., 2012; Liu et al., 2015), making it difficult to predict *a priori* which modifications may be important. Therefore, to quantify the concentration of each acyl-CoA species *in vivo*, we have developed a mass spectrometry (MS) probe that uses “native chemical ligation” (Dawson et al., 1994) to stably fix an acyl group from an acyl-CoA to the *N*-terminal amine of a cysteine residue via a thioester intermediate (CysTPP; Figure 1A). Furthermore, the fixed positive charge of a triphenylphosphonium (TPP) cation greatly enhances MS detection (Woo et al., 2009; Logan et al., 2014), allowing the quantification of *N-*acylated CysTPP in the low femtomole range. In addition to informing on the composition of the acyl-CoA pool, the reaction of CysTPP mimics the enhancement of protein lysine acylation caused by proximal cysteine thiols (Cohen et al., 2013; James et al., 2017, 2018a, 2018b; Hansen et al., 2019). Thus, the acyl modifications of CysTPP should reflect not only acyl-CoA concentrations but also their potential to cause non-enzymatic protein *N-*acylation *in vivo*.

Here, we use CysTPP to identify the acyl-CoAs that should be considered when assessing metabolic carbon stress *in vivo*. Critically, this work shows that hitherto unconsidered long-chain acyl-CoAs are abundant and can modify proteins. More generally, CysTPP should prove a useful tool for exploring other questions related to acyl-CoA pool composition.

## Results

### Design and synthesis of CysTPP

Native chemical ligation conjugates a peptide with an N-terminal cysteine residue to a second peptide with a C-terminal thioester (Dawson et al., 1994). This reaction is rapid, non-enzymatic and specific as it proceeds via a thioester intermediate on the thiol of the N-terminal cysteine residue. We reasoned that an acyl-CoA could substitute for the C-terminal thioester-containing peptide and that in the presence of an acyl-CoA an N-terminal cysteine-containing molecule could act as a probe by becoming irreversibly *N-*acylated. Furthermore, the remainder of the N-terminal cysteine containing peptide could be replaced with a triphenylphosphonium (TPP) cation, which has a fixed charge that greatly enhances detection by MS (Figure 1A) (Logan et al., 2014). Thus, we created [5-(2-amino-3-mercaptopropanoylamino)pentyl]triphenylphosphonium (CysTPP), a sensitive MS probe for detecting metabolites within biological extracts that are capable of acylating protein nucleophiles (Figure S1A).

CysTPP was synthesized as a pure stable disulfide precursor (CysTPP)_2_, in three steps from l-cystine (Figures S1A and S1B). This could then be activated by tris(2-carboxyethyl)phosphine (TCEP; Figures S1B and S2A), a reagent that reduces disulfides to thiols, but is nonreactive with thioesters (James et al., 2017).

### Reaction of CysTPP with acetyl-CoA

Incubation of CysTPP with acetyl-CoA and subsequent derivatization with iodoacetamide (IAM) led to the predicted *N-*acetylated and *S-*carbamidomethylated (CAM) product (*N-*acetyl-CysTPP-CAM) at 550 *m/z*, with its expected fragmentation pattern (Figures 1A and S2B). We chose the daughter ion peak at 459 *m/z* for multiple reaction monitoring (MRM) because it contains a molecular memory of the acetyl moiety from acetyl-CoA (Figures 1B and S2B). The presence of this 550→459 *m/z* mass transition is diagnostic for *N-*acetyl-CysTPP-CAM as the parent has additional mass equivalent to both acetyl and CAM moieties and the daughter has lost the mass of a CAM moiety plus an additional sulfur. As this 550→459 *m/z* transition likely arises through the fragmentation of the relatively weak C-S bond to give a dehydroalanine derivative, similar −91-Da neutral loss transitions can be used to identify other acylating species (e.g., palmitoyl-CysTPP-CAM will have a 746→655 *m/z* transition) (Figure S2B). Finally, pre-treatment of CysTPP with IAM prevents the formation of *N-*acetyl-CysTPP-CAM, demonstrating that the reaction largely proceeds via the native chemical ligation mechanism involving the rearrangement of an *S-*acetyl-CysTPP intermediate, and not by direct *N-*acetylation of the amine (Figure 1C).

Acyl groups can also be conjugated to carnitine via an ester bond *in vivo* and we have shown previously that *O-*acetyl-carnitine does not *N-*acetylate protein (James et al., 2017). Importantly, CysTPP does not react with *O-*acetyl-carnitine, and only weakly with *O-*acetyl-phosphate; thus, these esters will not contribute to *N-*acyl-CysTPP-CAM formation (Figure 1D). In contrast, CysTPP will react with other thioesters (Figure 1D); thus, it cannot differentiate between thioester acyl donors bearing the same acyl group, such as *S-*acetyl-CoA or *S-*acetyl-glutathione, if both are present.

CysTPP sequesters acetyl moieties with high efficacy, as the generation of *N-*acetyl-CysTPP-CAM saturates with a 2.5- to 5-fold excess of CysTPP over acetyl-CoA (Figure S1C). The rate constant of the reaction of 1 mM CysTPP with 200 μM acetyl-CoA to produce *N-*acetyl-CysTPP-CAM was 0.37 ± 0.03 M^−1^ s^−1^ (pH 7.8, 37°C). This is similar to the rate constants for intermolecular S→S acetyl transfer from acetyl-CoA to various thiols, which range from ∼0.05–0.5 M^−1^ s^−1^ (Bizzozero et al., 2001). Thus, the far slower S→N transfer (Figure 1C) ceases to be rate limiting because it is now an intramolecular reaction. This is consistent with peptide ligation by native chemical ligation (Dawson et al., 1994) and previous observations that cysteine thiols can enhance nearby lysine acylation on protein surfaces (Cohen et al., 2013; James et al., 2017, 2018a, 2018b; Hansen et al., 2019).

Thus, CysTPP reacts with acetyl-CoA to generate a stable product detectable by liquid chromatography with tandem mass spectrometry (LC-MS/MS).

### CysTPP reacts quantitatively with other acyl-CoAs

**Figure 2:**
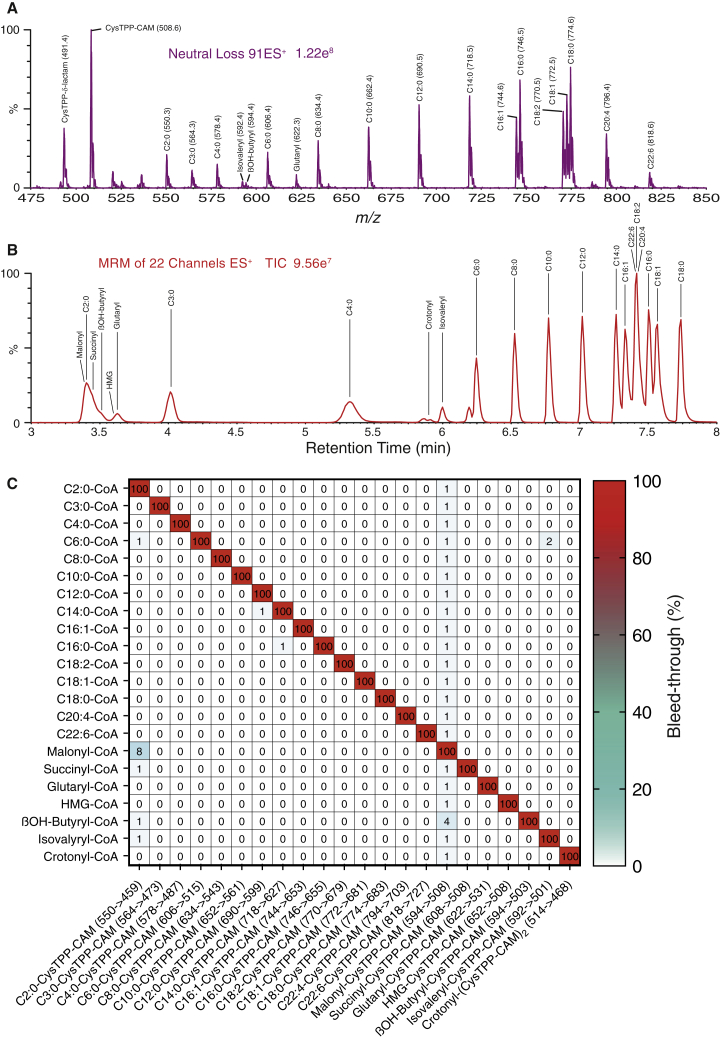
CysTPP reacts with a range of acyl-CoA standards to generate products that can be simultaneously detected by LC-MS/MS (A) Mixed solution of 22 acyl-CoA standards (5 μM of each) was reacted with 1 mM CysTPP followed by DTT and then IAM. This was solubilized in 80% (v/v) DMSO unless otherwise stated and acidified with 0.2% formic acid (FA). 18 acyl-CoA standards can be detected via neutral loss (−91 Da). (B) LC separation of 22 acyl-CoA standards. The gradient is isocratic 15% (v/v) ACN up to 5 min and then a linear gradient to 100% ACN (v/v) at 8 min. (C) Bleed-through of signal from 20 μM of an individual acyl-CoA standard into LC-MS/MS channels for all other *N-*acyl-CysTPP-CAM standard species. Values are the percentage of ion current in each channel for 20 μM of an individual acyl-CoA standard relative to the ion current in the channel for 20 μM of the acyl-CoA standard that should be detected by that channel. See also Figure S3.

Our main goal was to quantify the acylating species that proteins are exposed to in a particular tissue or organelle. Thus, CysTPP must react with a broad range of acyl-CoAs (see Figure S3A for nomenclature) present as a mixture to generate specific and stable products that can be separated and sensitively detected by LC-MS/MS. Individual reaction of 22 acyl-CoA standards with CysTPP led to 18 distinct neutral loss transitions of −91 Da (Figure 2A), the 4 exceptions being crotonyl-CoA and 3 carboxylate-CoA species (malonyl, succinyl and 3-hydroxy-3-methyl-glutaryl [HMG]). As an α,β-unsaturated carbonyl, crotonyl-CoA has two reactive centers and both can react with a CysTPP molecule to produce a crotonyl-(CysTPP-CAM)_2_ product (513.5 *m/z*; total mass 1,027). Fragmentation leads to a species at 468 *m/z* resulting from the neutral loss of −91 Da (Figure S3B). In contrast, malonyl-, succinyl-, and HMG-CoA all react to create the expected MS1 products, but fragmentation is mostly via decarboxylation to produce daughter ions at 508 *m/z* (Figure S3C).

**Figure 3:**
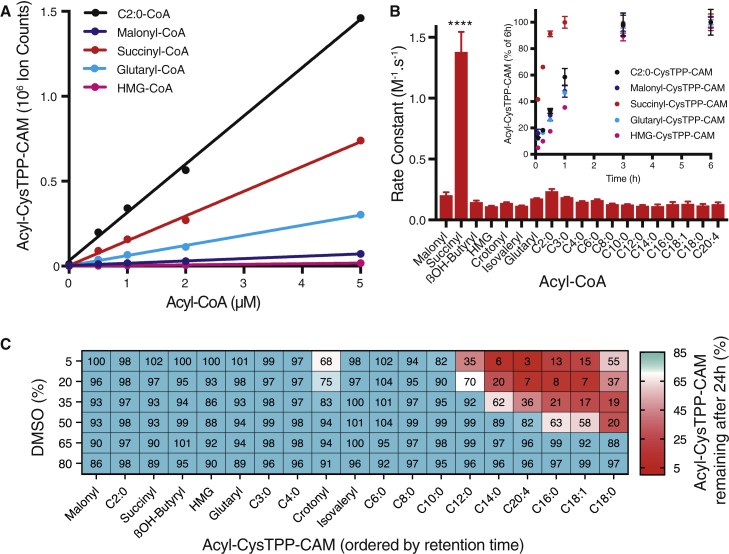
CysTPP sensitively and quantitatively detects a range of acyl-CoA standards (A) Generation of acyl-CysTPP-CAM is linear with acyl-CoA concentration. (B) Rate constants for the reaction of acyl-CoA with CysTPP. Inset, acyl-CysTPP-CAM generation ceases at 3 h. Data are the means ± SEMs (n = 3). Significance was calculated using a 1-way ANOVA and a Tukey multiple comparison test. ∗∗∗∗p < 0.0001. (C) Hydrophobic acyl-CysTPP-CAMs are lost from aqueous solution over time. Acyl-CysTPP-CAMs were solubilized in varying concentrations of DMSO, and their concentration was measured initially and again after 24 h at 8°C. See also Figure S4.

**Figure 4:**
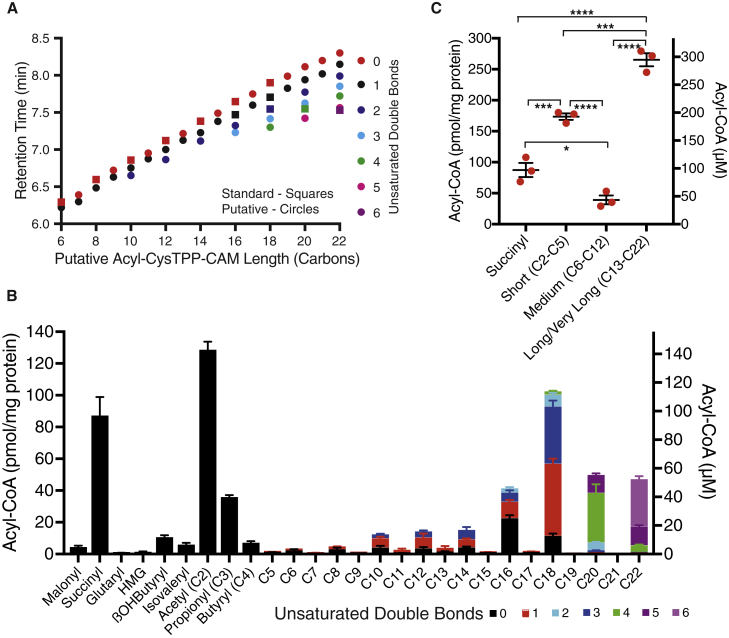
Acylating species in rat liver mitochondria (A) LC retention time and *m/*z can predict acyl-CysTPP-CAM species in the absence of acyl-CoA standards. Species with acyl-CoA standards are depicted as squares, while putative acyl-CoA species are shown as circles. (B) Abundance of acylating species in a crude fraction of isolated rat liver mitochondria extracted twice with 80% (v/v) methanol. Concentrations were calculated using standard curves for 22 acyl-CoAs reacted with CysTPP and assuming 0.9 μL mg protein^−1^. Data are from 3 independent mitochondrial preparations from 3 rats ± SEMs. (C) Cumulative concentration of long-chain (C13–C22) acylating species exceeds that of short, medium, and succinyl species. Data amalgamated from (B). Significance was calculated using a 1-way ANOVA and a Tukey multiple comparison test. ∗p < 0.05; ∗∗∗∗p < 0.001; ∗∗∗∗p < 0.0001. See also Figure S5 and Table S1.

**Figure 5:**
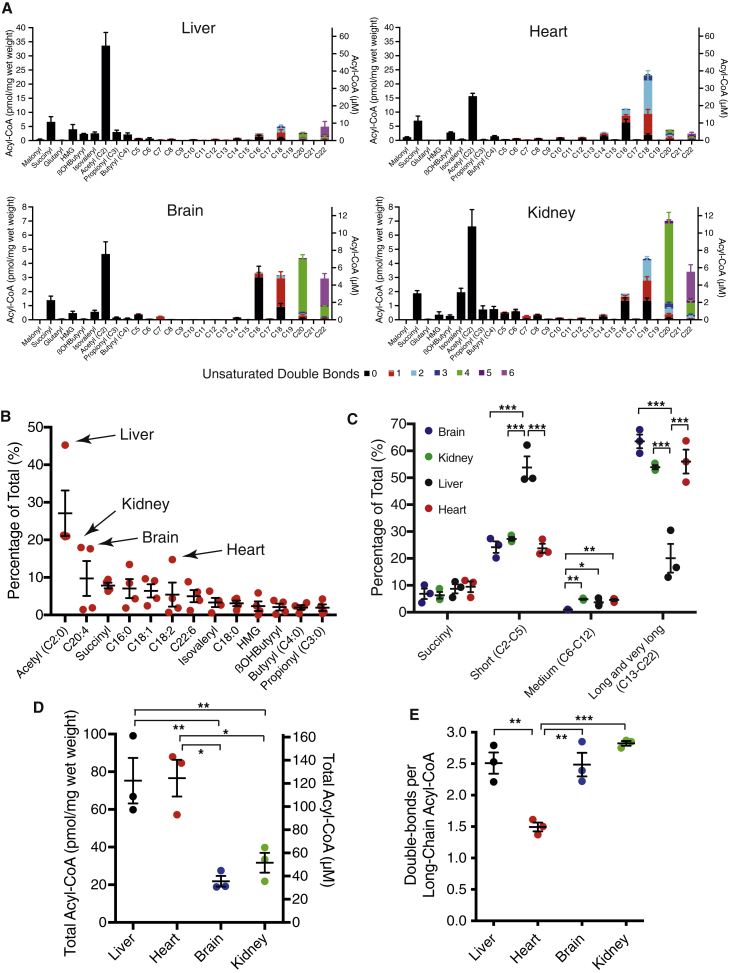
Acylating species in rat tissues Abundance of acylating species in snap-frozen rat tissue. Concentrations were calculated using standard curves for 22 acyl-CoAs reacted with CysTPP and assuming 0.615 μL mg wet weight of tissue^−1^. (A) Individual acylating species in liver, heart, kidney, and brain. Data are the mean from 3 rats ± SEMs. (B) The most abundant acylating species in liver, heart, kidney, and brain. Arrows point to abundant tissue-specific acyl-CoA species. Each data point is the mean of a tissue. Error bars show the means of all 4 tissues ±SEMs. (C) Acyl-CoA pool composition differs between tissues and long-chain acyl-CoAs are usually the most abundant class. Data are the mean from 3 rats ± SEMs. Significance was calculated using a 1-way ANOVA for each class of acyl-CoA and a Tukey multiple comparison test. ∗p < 0.05; ∗∗p < 0.01; ∗∗∗p < 0.001. (D) Total acyl-CoA concentrations vary between tissues. Data are the mean from 3 rats ± SEMs. Significance was calculated using a 1-way ANOVA and a Tukey multiple comparison test. ∗p < 0.05; ∗∗p < 0.01. (E) Acyl-CoA saturation varies between tissues. Data are the means ± SEMs (n = 3) of the average number of double bonds per long-chain (C13–C22) acyl-CoA in each tissue. Significance was calculated using a 1-way ANOVA and a Tukey multiple comparison test. ∗∗p < 0.01; ∗∗∗p < 0.001. See also Table S2.

Having identified transitions for each of the 22 acyl-CoA products, we optimized their LC separation (Figure 2B) to minimize the bleed through of signal between *m/z* channels (Figure 2C). This was generally ∼0.1%, and in all but two cases, it was <2% (Figure 2C). A small proportion of β-hydroxybutyryl-CysTPP-CAM fragmented via the 592→508 *m/z* transition used for quantifying malonyl-CysTPP-CAM (Figure 2C). In addition, there is some decarboxylation of malonyl-CysTPP-CAM that leads to minor contamination of the 550→459 *m/z* acetyl-CysTPP-CAM transition (Figure 2C). As malonyl-CoA concentrations are relatively low *in vivo* (see Figures 4 and 5), neither of these issues will affect the conclusions of this study. The formation of acyl-CysTPP-CAM products in a complex mixture was linear with concentration for all 22 acyl-CoA standards and a selection is shown in Figure 3A. Positively charged acyl-CysTPP-CAM products resulting from fatty acyl-CoAs displayed similar MS responses with a detection limit of ∼2 fmol of injected product (Table S1). The detection of most acyl-CysTPP-CAM species in positive ion mode appears to be more sensitive than the detection of their parental acyl-CoAs (Liu et al., 2015). However, the response of zwitterionic CysTPP-CAM products with carboxylic acid moieties was depressed in positive ion mode, with the detection limit of ∼5–50 fmol of injected product (Table S1). The combined acyl-CoA concentration (110 μM) was kept at less than the 200 μM acetyl-CoA concentration used for the optimization of the CysTPP concentration (1 mM; Figure S1C). The LC-MS/MS response was linear with concentration up to at least 25 pmol of an individual acyl-CoA when reacted and injected alone, or ∼1 pmol of each of 22 acyl-CoAs when reacted and injected together.

The reaction of CysTPP with all standards was prevented by pre-treatment with IAM (Figure S4A) and reached completion after 3 h at 37°C (a selection is shown in Figure 3B, inset). The rate constant of the reaction of 5 μM of 21 of the 22 acyl-CoAs with 1 mM CysTPP to produce 22 *N-*acyl-CysTPP products ranged from 0.11 to 0.24 M^−1^ s^−1^ at 37°C (Figure 3B). These values fall within the previously observed range (∼0.05–0.5 M^−1^ s^−1^) for the intermolecular S→S reaction of acetyl-CoA with various thiols (Bizzozero et al., 2001). The notable exception is succinyl-CoA, which has a significantly higher rate constant of 1.38 ± 0.17 M^−1^ s^−1^. This difference is likely a consequence of succinyl-CoA spontaneously generating a succinic anhydride intermediate (Wagner et al., 2017). This then reacts with the CysTPP thiolate as formation of succinyl-CysTPP-CAM was also prevented by the pre-treatment of CysTPP with IAM (Figure S4A).

The acyl-CysTPP-CAM products generated from the acyl-CoAs anticipated to be present *in vivo* elute from a conventional reversed phase-high-performance liquid chromatography (RP-HPLC) C18 column between 15% and 100% (v/v) acetonitrile (ACN; Figure 2B) as they differ widely in their hydrophobicity and solvent compatibility. Ideally, all acyl-CysTPP-CAM products would remain soluble in one solvent mixture in the MS autosampler to prevent bias toward the detection of a particular acyl-CoA. Therefore, we screened several solvents (methanol, ethanol, 2-propanol, 1-propanol, and dimethylformamide) in combination or alone, before selecting DMSO for its broad efficacy at solubilizing TPP cations. Finally, the DMSO concentration in water was varied to identify a concentration in which all acyl-CysTPP-CAMs were stable in solution over 24 h at 8°C. At DMSO concentrations >65%, the more hydrophobic acyl-CysTPP-CAM products remained in solution over 24 h (Figures 3C and S4B–S4D). In contrast, at DMSO concentrations <65%, the detection of more hydrophobic acyl-CysTPP-CAM products greatly diminished after 24 h. Thus, CysTPP can quantitatively detect low fmol amounts of a range of acyl-CoA standards.

### CysTPP detects acyl-CoAs extracted from rat liver mitochondria

To assess whether CysTPP could quantitatively detect a range of acyl-CoAs extracted from a biological sample, we extracted metabolites from isolated rat liver mitochondria, as they have a much higher acyl-CoA concentration than the cytosol (Wagner and Payne, 2013). In neutral loss scans of trial extracts, several additional −91 *m/z* shifts characteristic of acyl-CysTPP-CAM species could be seen. Using MS parameters optimized for the original 19 acyl-CysTPP-CAM products for which we had acyl-CoA standards, systematic transitions were established for monitoring 44 additional acyl-CysTPP-CAM products. The probable carbon length and saturation of these putative acyl-CysTPP-CAM products correlated well with the retention times of our initial 19 acyl-CoA standards (Figure 4A). Three of the prominent putative candidates present in mitochondria were subsequently confirmed by comparison of their retention times with additional acyl-CoA standards (C16:1, C18:2, and C22:6).

To represent the acyl-CoA population occurring in a tissue *in vivo* without bias, extraction needs to be equivalent for a broad range of acyl-CoA species. After screening several solvents at different concentrations, two sequential extractions with 80% (v/v) methanol were the most effective at extracting a broad range of acyl-CoAs to a similar extent (Figure S5A). A third extraction with 80% (v/v) methanol only recovered an additional 5%–10%. While some solvents were marginally better for individual acyl-CoA species, they had major inefficiencies with certain classes of acyl-CoA (Figure S5B). Furthermore, this cold methanol extraction precipitates protein preventing *S-*acyl cysteine residues from contributing to the acyl-CysTPP-CAM signal.

Each of the 22 acyl-CysTPP-CAM peaks with available standards was quantified relative to a standard curve of known acyl-CoA concentration (0-5 μM in the reaction with CysTPP; 0–1.2 pmol injected into the LC-MS/MS). Of the standards, only crotonyl-CoA had no detectable signal in extracts. To quantify the remaining 41 species lacking a standard, the standard closest in properties was used (e.g., the C16:1 standard was used for C16:2; Table S1). The fatty acyl-CysTPP-CAM peaks without standards will have LC-MS/MS properties similar to the closest standards as there was little variation in MS response between similar neutral fatty acyl-CysTPP-CAM standards (Table S1). The 22 standards allowed direct quantification of all non-fatty-acyl species and 82.3% ± 1.7% of the overall acyl-CoA population by concentration in isolated rat liver mitochondria (Figure 4B). In most cases, the detection limit is ∼2 fmol of injected acyl-CysTPP-CAM, and 50 of the 63 putative species were present at a concentration exceeding this. This limit equates to a mitochondrial matrix acyl-CoA concentration of ∼0.5 μM, assuming a matrix volume of 0.9 μL mg protein^−1^ (Halestrap, 1989). While detection using a TPP cation appears to be relatively sensitive for most acyl-CoAs, the low abundance and negative charge of malonyl-, HMG-, and glutaryl-CoA may make them better suited to direct acyl-CoA measurements. Succinyl-CoA is an exception to this as it is abundant and its higher reactivity (Figure 3B) makes it less stable in extracts (Liu et al., 2015).

This instability is caused by anhydride formation (Wagner et al., 2017) and other species present in biological extracts that can affect the acyl-CoAs concentrations measured. Measurement of many acyl-CoAs directly with LC-MS/MS using acyl-CoA fragments will be affected by even higher tissue concentrations of glutathione that lead to the loss of acyl-CoA signal due to *S-*acyl-glutathione formation (Liu et al., 2015; James et al., 2017). Measurement of acyl-CoAs with CysTPP avoids this as *S-*acyl-glutathiones generated from acyl-CoAs post-extraction irreversibly form the same stable acyl-CysTPP-CAM product (Figure 1D and the Discussion). However, using CysTPP to measure acyl-CoAs requires an incubation at 37°C that is not needed when acyl-CoAs are measured directly by LC-MS/MS. This step could allow iron and copper that is released in the extraction to subsequently oxidize unsaturated acyl-CoAs. The inclusion of 100 μM diethylenetriaminepentaacetic acid (DTPA), 100 μM neocuproine, or 1 mM butylated hydroxytoluene (BHT) in the extraction solution prevented the formation of the lipid peroxidation product hydroxynonenal (HNE; Figure S5C), but did not increase the detection of unsaturated fatty acyl-CoAs (Figure S5D). Furthermore, although the data shown here were from samples analyzed by LC-MS/MS immediately after derivatization, the acyl-CysTPP-CAM products were stable for at least several days (Figures S5E and S5F).

The combined concentration of the even-length fatty acyl-CysTPP-CAM peaks (excluding acetyl) is 24-fold greater than that of the odd-length fatty acyl-CysTPP-CAM peaks (excluding propionyl), consistent with the expected *in vivo* profile (Figure 4B). Critically, for this work, the combined concentrations of longer-chain acyl-CoAs (C13-C22) is high (296 ± 12 μM) and exceeds that of acetyl-CoA (143 ± 5 μM) and succinyl-CoA (97 ± 13 μM; Figure 4C). Together, these hydrophobic acyl-CoAs represent 45.1% ± 0.9% of the total acyl-CoA pool in liver mitochondria (656 ± 13 μM). Acyl-CoAs longer than C16 do not undergo β-oxidation in the mitochondrial matrix, and their presence likely reflects a bona fide mitochondrial outer membrane pool for phospholipid synthesis (Yu et al., 2018), as well as contaminating endoplasmic reticulum (ER) and peroxisomal pools.

### CysTPP can also detect acyl-CoAs in tissue extracts

Although isolated mitochondria contain high concentrations of acyl-CoAs, making proof of concept detection easier, it is possible acyl-CoA concentrations may change during isolation. To avoid this and determine acyl-CoA concentrations *in vivo*, we snap froze rat heart, liver, kidney, and brain tissue in liquid N_2_ and extracted ∼20 mg of tissue twice with 80% (v/v) methanol. These extracts were reacted with CysTPP, derivatized with IAM, and 63 acyl-CysTPP-CAM transitions were assessed. The acyl-CoA standards allowed direct quantification of all of the non-fatty-acyl species and 85.7%–92.2% of the overall acyl-CoA population in the four tissues (Figure 5A; Table S2).

To enable comparison with isolated mitochondria and other metabolites, tissue concentrations were also calculated using an intracellular water content value from heart tissue with a wet weight of 0.615 μL mg^−1^ (Aliev et al., 2002). This should also provide a reasonable approximation of the acyl-CoA concentration in brain, liver, and kidney. However, the amphipathic nature of some acyl-CoAs as well as compartmentation of the metabolic reactions they are involved in will mean cellular distribution is probably very uneven. Consequently, local concentrations within cells could vary significantly from those shown (Figure 5A). For example, the relative concentrations of succinyl-CoA in liver tissue (10.9 μM) and isolated liver mitochondria (97 μM) and the mitochondrial volume fraction of liver tissue of 0.1–0.14 (Krahenbuhl et al., 1992) are consistent with succinyl-CoA having an exclusively mitochondrial location. Hence, its compartmentation within the small matrix volume and its 5-fold higher rate constant (Figure 3B) imply significant non-enzymatic succinylation within mitochondria even though it represents only 7.9% ± 0.7% of the acyl species in all 4 tissues (Figure 5B). Reassuringly, the relative concentration of reactive succinyl-CoA in tissue when compared with mitochondria (0.112) is equivalent to the mitochondrial volume fraction (0.1–0.14), suggesting that mitochondrial isolation has not had a major impact on our measured mitochondrial acyl-CoA concentrations (Figure 4).

Our value for the acetyl-CoA concentration in rat liver determined using CysTPP (35 nmol g tissue^−1^) is comparable to a previous direct measurement of acetyl-CoA by LC-MS/MS in mouse liver of 30–40 nmol g tissue^−1^ (Palladino et al., 2012). It has been tacitly assumed that acetyl-CoA is the major non-enzymatic *N-*acylating species *in vivo* partly because of its many roles in metabolism. While this is true for the frequently studied liver, where 45% ± 2% of the acylating species acetyl-CoA greatly exceeds all others, our work shows it is an oversimplification for other tissues (Figure 5A). In heart, brain, and kidney it remains the single largest species, but accounts for only 21% of the acyl-CoA species we quantify. The acyl-CoA pool of these tissues is dominated by longer-chain (C13–C22) acyl-CoAs, with this group representing ∼60% of all acyl-CoAs (Figure 5C). For example, in the brain, the concentration of arachidonyl-CoA alone (C20:4; 4.8 ± 0.3 μM) is almost equivalent to that of acetyl-CoA (5.8 ± 1.0 μM). In addition, the composition of the longer-chain (C13–C22) fatty acyl-CoA pool differs substantially between tissues. While each tissue contains considerable quantities of palmitoyl-CoA (C16:0) and oleoyl-CoA (C18:1), in the heart, linoleoyl-CoA (C18:2) is abundant, and in the brain and kidney, arachidonyl-CoA (C20:4) and docosahexaenoyl-CoA (C22:6) are common (Figure 5B). While short- and long-chain acyl-CoA species are relatively abundant, the concentrations of individual medium-chain (C6, C8, C10, and C12) species are comparatively low in liver, heart, and kidney (0.2–1 μM; Figures 5A and 5C). Interestingly, in brain tissue, the concentration of these medium-chain acyl-CoAs are a further 10- to 100-fold lower, making them effectively undetectable. Apart from acetyl and succinyl, many of the acyl modifications to protein previously identified by LC-MS/MS after antibody enrichment arise from acyl-CoA species that are present at very low concentrations (Figure 5B; Table S2). In the absence of a specific acyltransferase or a selective interaction, it is difficult to envision regulation by these reported modifications as they are only a small percentage of the acyl-CoA pool and thus must compete for acylation sites with other, far more abundant acyl-CoAs.

For protein acylation, absolute acyl-CoA concentrations are important and both heart and liver have 3- to 4-fold greater concentrations of acylating species than either brain or kidney (Figure 5D). Finally, unsaturated double bonds may affect the behavior of acyl modifications—for example, by providing additional reactive centers that could oxidize or generate cross-links with other protein residues (Figure S5). In this regard, it is interesting to note that heart long-chain acyl-CoAs are significantly less saturated than liver, kidney, or brain (Figure 5E).

In summary, we have successfully developed a novel LC-MS/MS assay that sensitively detects and quantifies a wide range of acyl-CoAs in tissue. Importantly, the chemical mechanism by which CysTPP derivatizes acyl-CoAs supports the idea that they could all modify nucleophilic protein residues (Cohen et al., 2013; James et al., 2017, 2018a; Hansen et al., 2019).

### Long-chain acyl-CoAs can acylate protein residues *in vitro*

**Figure 6:**
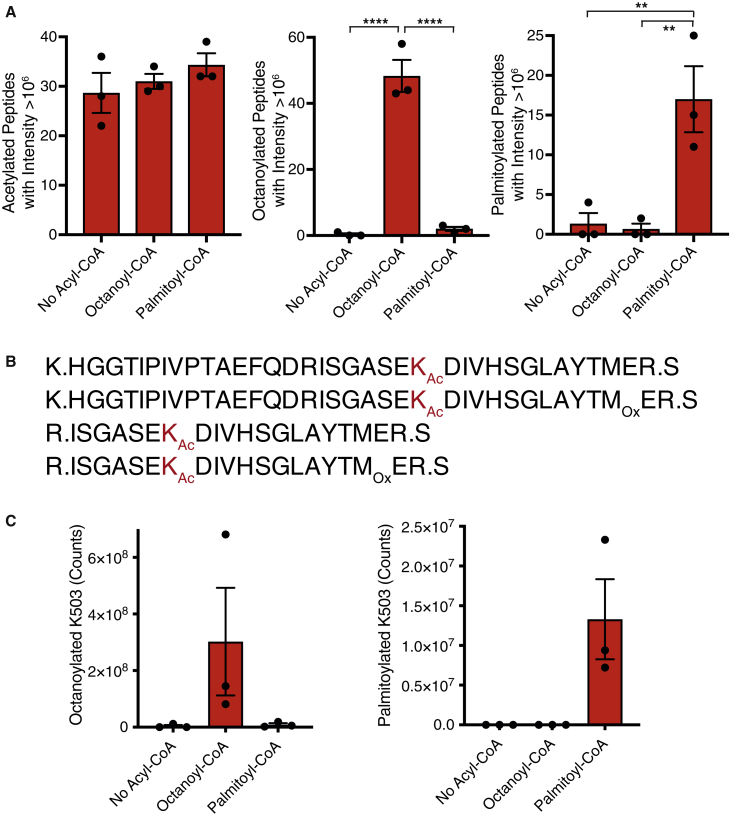
Medium- and long-chain acyl-CoAs acylate protein Purified bovine liver GDH was treated with nothing or 2 mM of either octanoyl-CoA or palmitoyl-CoA for 6 h at 37°C. GDH was precipitated, with the resulting pellet trypsinized and resuspended in 45% ACN for LC-MS/MS. (A) Purified GDH is already acetylated and can be further acylated by octanoyl-CoA and palmitoyl-CoA *in vitro*. Data are the mean number of unique acetylated, octanoylated, or palmitoylated peptides with an intensity >10^6^ identified by LC-MS/MS ± SEM (n = 3). Significance was calculated using a 1-way ANOVA and a Dunnett’s multiple comparison test. ∗∗p < 0.01; ∗∗∗∗p < 0.0001. (B) Acylation of K503 (K_Ac_, red) is detected on 4 tryptic peptides due to miscleavage and methionine oxidation (M_Ox_). (C) K503 is either octanoylated or palmitoylated when incubated with either octanoyl-CoA or palmitoyl-CoA, respectively. Data are the sum of the area of the 4 acylated peptides in (B). Data are the means ± SEMs (n = 3). See also Figure S6 and Table S3.

The focus of this paper was to develop an MS probe and analytical approach that could characterize the acyl-CoA species present *in vivo* to identify abundant potential acylating species that may modify protein residues *in vivo*. We have accomplished this primary goal and identified high concentrations of long-chain acyl-CoAs in tissues that could modify nucleophilic residues, such as cysteine and lysine, on the surface of proteins. That these modifications will occur on protein is likely as CysTPP chemically mimics a lysine residue with a catalytic cysteine residue nearby (Cohen et al., 2013; James et al., 2017, 2018a; Hansen et al., 2019). Nevertheless, it remained important to demonstrate that the non-enzymatic reaction of long-chain acyl-CoAs with proteins could occur. Treatment of bovine glutamate dehydrogenase (GDH) with malonyl-CoA leads to *N-*malonylation of lysine residues, particularly at K503 (James et al., 2020). To demonstrate that medium and long-chain acyl-CoAs can also non-enzymatically *N-*acylate protein, we incubated GDH with either octanoyl-CoA or palmitoyl-CoA for 6 h at 37°C and detected acylated peptides by LC-MS/MS. We observed widespread *N-*octanoylation or *N-*palmitoylation, respectively, of many peptides from GDH in response to exogenous acyl-CoAs, with no effect on the amount of endogenously *N-*acetylated peptides detected (Figure 6A; Table S3). More specifically, the *N-*octanoylation or *N-*palmitoylation of tryptic peptides containing K503 (Figures 6B and S6) was elevated when either octanoyl-CoA or palmitoyl-CoA, respectively, were present (Figure 6C; Table S3).

Thus, long-chain acyl-CoAs can non-enzymatically cause long-chain acyl modification of lysine residues *in vitro*. Our application of CysTPP to tissue extracts indicates the mass shifts on proteins that should be targeted when assessing whether non-enzymatic acylation of proteins is important for pathology.

## Discussion

Despite the centrality of acyl-CoAs to metabolism, there is surprisingly little information on the relative makeup of the acyl-CoA pool, particularly in different tissues. Many studies consider only certain classes of acyl-CoAs and often only in a single tissue with a metabolic focus (Deutsch et al., 1994; Blachnio-Zabielska et al., 2011; Palladino et al., 2012; Liu et al., 2015). This constrained our ability to predict likely protein modifications to target with proteomics. Here, we have developed CysTPP, an MS probe that traps activated acyl groups within tissue samples as stable species that can be readily quantified. All acyl-CoAs tested transferred their acyl moiety to the amine of CysTPP via its thiol, and, with the exception of succinyl-CoA, all were equally reactive (Figure 3B). This thiol-dependent mechanism of CysTPP intentionally mimics proximity-dependent non-enzymatic protein *N-*acylation of surface lysine residues observed *in vitro* (Cohen et al., 2013; James et al., 2017, 2020) and *in vivo* (Cohen et al., 2013; James et al., 2018a, 2018b, 2020; Hansen et al., 2019). Thus, if non-enzymatic protein acylation contributes to carbon stress *in vivo*, the most abundant acyl groups identified by CysTPP should reflect the non-enzymatic modifications of protein residues to be sought *in vivo* to test this hypothesis. If certain modifications at specific sites are more abundant than the acyl-CoA profile suggests (Figures 4 and 5), then it may indicate the action of acyl-transferases specific for individual acyl-CoAs and proteins.

For the most part, the acyl groups trapped by CysTPP will derive from the acyl-CoA pool and reflect the acyl-CoAs that are measured directly by LC-MS/MS (Liu et al., 2015). However, acyl-CoAs are unstable, particularly in the presence of other thiols, and this will affect and limit each technique differently. While the majority of donors *in vivo* will be *S-*acyl-CoAs as these are directly generated by metabolism, some *S-*acyl-glutathione may exist in equilibrium with its *S-*acyl-CoA before it is hydrolyzed by hydroxyacylglutathione hydrolase (James et al., 2017). Furthermore, cyclic anhydrides may spontaneously form from some acyl-CoAs that contain carboxylates (Wagner et al., 2017). Thus, measurement of acyl-Co-As by CysTPP relies on a relatively low *in vivo* concentration of acyl-glutathione and anhydrides and this may not always be true. Equally, as glutathione is present at a greater concentration than total acyl-CoA pool *in vivo*, there may be appreciable acyl-transfer from acyl-CoAs to excess glutathione during sample preparation, storage, and analysis of tissue extracts even at 4°C (Liu et al., 2015; James et al., 2017). Anhydrides could also form in samples. Any *S-*acyl-glutathiones and anhydrides that are generated will no longer be measured as acyl-CoAs by LC/MS/MS that directly targets acyl-CoAs and their fragment ions (Liu et al., 2015). In contrast, extracted *S-*acyl-CoAs, as well as the *S-*acyl-glutathiones and anhydrides that form from acyl-CoA breakdown in samples, will irreversibly form the same stable acyl-CysTPP-CAM product when CysTPP is used (Figure 1D). Critically, this leads to similar stability for standards and samples (Figures S5E and S5F), thereby enabling the generation of absolute acyl-CoA concentrations using a large number of simple standard curves rather than a few spiked heavy isotope-labeled acyl-CoAs (Blachnio-Zabielska et al., 2011; Palladino et al., 2012). Thus, CysTPP allows a relative comparison of the *in vivo* concentration of all of the main acyl-CoA species, thereby enabling a complete picture of the acyl-CoA pool. This absolute quantification is also important for evaluating the carbon stress hypothesis as it allows comparison of acyl-CoAs with other more reactive, but much less abundant “stress” species, such as reactive oxygen species (ROS), that may damage protein.

Across the four tissues analyzed here, the most abundant acyl-CoA species were acetyl-CoA, arachidonyl-CoA (C20:4), succinyl-CoA, palmitoyl-CoA (C16:0), oleoyl-CoA (C18:1), linoleoyl-CoA (C18:2), and docosahexaenoyl-CoA (C22:6). The abundant long-chain acyl-CoAs species identified by CysTPP were consistent with those identified directly by conventional metabolomics (Deutsch et al., 1994; Blachnio-Zabielska et al., 2011; Palladino et al., 2012; Liu et al., 2015). While acetyl-CoA is abundant in frequently studied liver, absolute quantification of all acyl-CoAs simultaneously in other tissues indicates that hydrophobic longer-chain acyl-CoAs are a large proportion (∼60%) of the acyl groups attached to the CoA pool *in vivo* (Figure 5C). We demonstrated the plausibility of non-enzymatic reactions of longer-chain acyl-CoAs with protein by treating a single protein with palmitoyl-CoA *in vitro* (Figure 6). Consistent with previous work with acyl-CoAs *in vitro* (Wagner and Payne, 2013; James et al., 2017, 2020), we observe *N-*acyl modification of many surface lysine residues. However, these modifications will be difficult to observe in complex protein extracts. First, the median stoichiometry of non-enzymatic acetylation is only ∼0.1% (Weinert et al., 2015; Hansen et al., 2019); consequently modified peptides are usually detected after they have been enriched with antibodies. No antibodies exist for any of the ∼40 modifications generated by hydrophobic acyl-CoAs. Second, even if long-chain acyl modifications cumulatively represent a significant problem on the surface of proteins, the individual stoichiometry of each long-chain acyl modification will be relatively low (Figures 4 and 5). Finally, detection of these long-chain acyl modifications will require sample preparations to be altered so unsaturated modifications are not oxidized, the acylated peptide remains soluble, as well as the modification to be anticipated and included in software search parameters. Consequently, the ∼40 medium- and long-chain acyl modifications suggested by this work are a technical blind spot for current proteomic approaches that rely on antibody enrichment, aqueous solvents, and *a priori* knowledge.

Modification of a lysine residue by hydrophobic long-chain acyl-CoAs both neutralizes its charge and greatly enhances its hydrophobicity. Future work will focus on the extent to which low-stoichiometry modification of protein residues by long-chain acyl groups occurs *in vivo* and then determine whether they can disrupt protein homeostasis, potentially contributing to the many pathologies associated with protein aggregation.

### Limitations of the study

Here, we measure acyl-CoAs in tissues by trapping their acyl moiety with CysTPP. While this allows stable and sensitive detection of acyl-CoAs, there are caveats as CysTPP will also accept acyl groups from other donors if they are present. Although acyl-CoAs will be the main acyl donors to CysTPP *in vivo* as they are generated directly by metabolism, acyl-glutathiones, anhydrides, and acyl groups on protein cysteine, residues can form from acyl-CoAs and will generate identical acyl-CysTPP-CAM products. These could contribute to the acyl-CoA signal that is detected if they are present at significant quantities. In this study, the contribution of acyl groups from cysteine residues to the reported acyl-CoA concentration was limited by protein precipitation, but in the future using a modified assay, this population of acyl groups could also be explored with CysTPP.

### Summary

This work develops CysTPP, a highly sensitive LC-MS/MS probe that selectively traps thioester-bound acyl groups using chemistry akin to native chemical ligation. It advances our understanding of the composition of the acyl-CoA pool, demonstrates the broad range of acylating species *in vivo* and identifies key modifications that may occur on the surface of proteins. In particular, long-chain acyl-CoAs represent a large proportion of the acyl-CoAs in many of the tissue extracts we assessed here, and their hydrophobic properties are likely to alter the behavior of proteins with which they react.

## STAR★Methods

### Key resources table

**Table:** 

REAGENT or RESOURCE	SOURCE	IDENTIFIER
**Chemicals, peptides, and recombinant proteins**		

(CysTPP)_2_⋅2Cl⋅2HCl = disulfide of [5-(2(R)-amino-3-mercaptopropanoylamino)pentyl]triphenylphosphonium chloride, bis-hydrochloride salt	This paper	N/A
Iodoacetamide (IAM)	Sigma-Aldrich	Cat#I1149; CAS#144-48-9
Glutamate Dehydrogenase - bovine liver	Sigma-Aldrich	Cat#G7882; CAS#9029-12-3
Pierce™ TCEP-HCl	ThermoFisher	Cat#20490; CAS#51805-45-9
Acetyl-CoA sodium salt	Sigma-Aldrich	Cat#A2056; CAS#102029-73-2
Malonyl-CoA lithium salt	Sigma-Aldrich	Cat#P5397; CAS#108347-84-8
Propionyl-CoA lithium salt	Sigma-Aldrich	Cat#P5397; CAS#1008321-21-7
Succinyl-CoA lithium salt	Sigma-Aldrich	Cat#S1129; CAS#108347-97-3
DL-3-hydroxybutyryl-CoA lithium salt	Sigma-Aldrich	Cat#H0261; CAS#103404-51-9
Crotonoyl-CoA trilithium salt	Sigma-Aldrich	Cat#28007
Butyryl-CoA lithium salt hydrate	Sigma-Aldrich	Cat#B1508
Glutatryl-CoA lithium salt	Sigma-Aldrich	Cat#G9510; CAS#103192-48-9
Isovaleryl-CoA lithium salt hydrate	Sigma-Aldrich	Cat#I9381
DL-3-hydroxy-3-methylglutaryl-CoA lithium salt	Sigma-Aldrich	Cat#H6132; CAS#103476-21-7
Hexanoyl-CoA trilithium salt	Sigma-Aldrich	Cat#H2012; CAS#103476-19-3
Octanoyl-CoA lithium salt hydrate	Sigma-Aldrich	Cat#O6877; CAS#324518-20-9
Decanoyl-CoA monohydrate	Sigma-Aldrich	Cat#D5269; CAS#1264-57-9
Lauroyl-CoA lithium salt	Sigma-Aldrich	Cat#L2659; CAS#190063-12-8
Myristoyl-CoA lithium salt	Sigma-Aldrich	Cat#M4414; CAS#187100-75-0
Palmitoleoyl-CoA lithium salt	Sigma-Aldrich	Cat#P6775; CAS#18198-76-0
Palmitoyl-CoA lithium salt	Sigma-Aldrich	Cat#P9716; CAS#188174-64-3
18:2-(n6)-CoA ammonium salt	Avanti	Cat#870736P; CAS#1246304-39-1
Oleoyl-CoA lithium salt	Sigma-Aldrich	Cat#O1012; CAS#188824-37-5
Stearoyl-CoA lithium salt	Sigma-Aldrich	Cat#S0802; CAS#193402-48-01
Arachidonyl-CoA lithium salt	Sigma-Aldrich	Cat#A2056; CAS#188174-63-2
22:6-CoA ammonium salt	Avanti	Cat#870728P; CAS#800377-20-2
*O*-acetyl-L-carnitine HCl	Sigma-Aldrich	Cat#A6706; CAS#5080-50-2
Acetyl-phosphate Li/K salt	Sigma-Aldrich	Cat#A0262; CAS#94249-01-1
*S-*acetyl-glutathione	Iris Biotech, Germany	Cat#LS-1270; CAS#3054-47-5
4-hydroxynonenal (HNE)	Cayman Chemical	Cat#32100 CAS#75899-68-2
Anhydrous 1,4-dioxane	Sigma-Aldrich	Cat# 296309-1L CAS# 123-91-1
Anhydrous *N,N*-dimethylformamide	Sigma-Aldrich	Cat# 227056-1L CAS# 68-12-2
CDCl_3_	Cambridge Isotope Laboratories, Inc	Cat# DLM-7-100MS CAS# 865-49-6
DMSO-*d*_*6*_	Cambridge Isotope Laboratories, Inc	Cat# DLM-10-10X0.75 CAS# 2206-27-1
Concentrated aqueous hydrochloric acid	Honeywell	Cat# 07102-2.5L CAS# 7647-01-0
L-Cystine	Alfa-Aesar	Cat# A13762 CAS# 56-89-3
Di-*tert*-butyl dicarbonate	Fluorochem	Cat# 021896 CAS# 24424-99-5
*N,N*-Diisopropylethylamine	Fluorochem	Cat# 005027 CAS# 7087-68-5
Boc-L-cystine	This paper	N/A
*N,N,Nʹ,Nʹ*-Tetramethyl-*O*-(1*H*-benzotriazol-1-yl)uronium hexafluorophosphate	Flurochem	Cat# 009019 CAS# 94790-37-1
(5-Aminopentyl)triphenylphosphonium bromide hydrobromide	This paper	N/A
(Boc-CysTPP)_2_•2Cl = disulfide of {5-[3-mercapto-2(*R*)-(*tert*-butoxycarbonylamino)propanoylamino]pentyl}triphenylphosphonium chloride	This paper	N/A

**Critical commercial assays**		

Pierce™ BCA Protein Assay Kit	ThermoFisher	Cat#23225

**Experimental models: Organisms/strains**		

Female Wistar rats	Charles River	N/A

**Software and algorithms**		

GraphPad Prism 9	GraphPad Software	N/A
PEAKS X	Bioinformatics Solutions Inc.	Version 10.6 build 20201221
MassLynx 4.1	Waters	N/A

**Other**		

ACQUITY UPLC® BEH C18 MS Column (1.7 μm, 130 Å, 50 × 1 mm)	Waters	Cat#186002344
Precellys24 tissue homogenizer	Bertin Instruments	N/A
Tissue lysis tubes with 2.8 mm ceramic beads	Omni	N/A
Eppendorf Protein LoBind tubes 1.5 mL	Eppendorf	Cat#022431081
ACQUITY UPLC® I-Class	Waters	N/A
Xevo TQ-S mass spectrometer	Waters	N/A
Q-Exactive Plus mass spectrometer	Thermo Fisher Scientific	N/A
RSLC 3000 nanoUPLC	Thermo Fisher Scientific	N/A
PepMap RSLC C18 EASYspray column (2 μm, 100 Å, 75 μm × 50 cm)	Thermo Fisher Scientific	P/N ES803A
MS vials	Waters	Cat#186005662CV

### Resource availability

#### Lead contact

Michael P. Murphy (mpm@mrc-mbu.cam.ac.uk).

#### Materials availability

This study generated the following unique reagent: (CysTPP)_2_

This reagent is available from MPM or RCH (Richard.Hartley@glasgow.ac.uk) under Materials Transfer Agreements.

### Experimental model and subject details

#### Animals

All experiments were carried out in accordance with the UK Animals (Scientific Procedures) Act of 1986 and the University of Cambridge Animal Welfare Policy. Wistar rats (003, wildtype, female) were ordered from Charles River Laboratories UK (Margate, UK). They were housed under standard laboratory conditions with ad lib food and water and used between 10-12 weeks. Rats were culled by cervical dislocation with accordance to UK Home Office Schedule 1.

### Method details

#### Synthesis of (CysTPP)_2_

(CysTPP)_2_ was prepared from L-cystine in three steps (Figure S1A). The amino groups of L-cystine were protected as *tert*-butyl carbamates to give Boc-L-cystine in good yield. Coupling of the free carboxylic acids with (5-aminopentyl)triphenylphosphonium chloride using *N,N,Nʹ,Nʹ*-tetramethyl-*O*-(1*H*-benzotriazol-1-yl)uronium hexafluorophosphate (HBTU), followed by ion exchange gave (Boc-CysTPP)_2_ as the bischloride salt. Deprotection with acid gave (CysTPP)_2_ with its amino groups protonated as the tetrachloride salt, (CysTPP)_2_•2Cl•2HCl. Full synthetic details are presented below. ^1^H NMR spectra in DMSO-*d*_*6*_ are referenced to residual protons of partially deuterated solvent at d 2.50 and ^13^C NMR spectra to the solvent peak at d 39.52 (Fulmer et al., 2010). All spectra are fully assigned: where needed atom numbering is used and this corresponds to the name; C-P coupling constants were used to assign the signals from ^13^C nuclei *ortho* and *meta* to phosphorus in the ^13^C NMR spectrum (Gray, 1973). Raw data and processed spectra can be found at 10.5525/gla.researchdata.1214.

##### Disulfide of 3-mercapto-2(R)-(tert-butoxycarbonylamino)propanoic acid, Boc-L-cystine

To an ice-cooled solution of L-cystine (5.00 g, 20.8 mmol, 1.00 eq) in deionized water (100 mL) was added sodium carbonate (8.80 g, 83.2 mmol, 4.00 eq) followed by the dropwise addition of a solution of di-*tert*-butyl dicarbonate (13.6 g, 62.4 mmol, 3.00 eq) in 1,4-dioxane (50.0 mL). The reaction mixture was allowed to stir at room temperature for 24 h then cooled to 0°C. The solution was acidified to pH 4 with the dropwise addition of concentrated aqueous hydrochloric acid then extracted with ethyl acetate. The combined organic layers were washed with brine, dried over anhydrous magnesium sulfate and concentrated under reduced pressure to give Boc-L-cystine as a white amorphous solid (7.49 g, 82%). δ_H_ (400 MHz, DMSO-*d*_*6*_): 1.37 (18H, s, 6 × CH_3_), 2.87 (2H, dd, *J* = 13.8, 10.3 Hz, 2 × C*H*^*A*^H^B^S), 3.10 (2H, dd, *J* = 13.8, 4.4 Hz, 2 × CH^A^*H*^*B*^S), 4.16 (2H, apparent td, *J* = 9.4 and 4.3 Hz, 2 × C*H*NHBoc), 7.18 (2H, d, *J* = 8.8 Hz, 2 × NH), 12.90 (2H, br s, 2 × OH); δ_C_ (101 MHz, DMSO-*d*_*6*_): 28.2 (CH_3_), 39.4 (CH_2_), 52.7 (CH), 78.3 (C of ^t^Bu), 155.4 (carbamate C=O), 172.4 (carboxylic acid C=O); HRMS (ESI^+^, m/z): found [M+Na]^+^ 463.1183. C_16_H_28_N_2_NaO_8_S_2_^+^ requires 463.1179. NMR data assignment assisted by HSQC. Spectral data agree with the literature (Zheng et al., 2017).

##### Disulfide of {5-[3-mercapto-2(R)-(tert-butoxycarbonylamino)propanoylamino] pentyl}triphenylphosphonium chloride, (Boc-CysTPP)_2_•2Cl

To a solution of the disulfide of 3-mercapto-2(*R*)-(*tert*-butoxycarbonylamino)propanoic acid (0.566 g, 2.36 mmol, 1.00 eq) in anhydrous *N,N*-dimethylformamide (20.0 mL) in a flame-dried flask under argon was added *N,N,Nʹ,Nʹ*-tetramethyl-*O*-(1*H*-benzotriazol-1-yl)uronium hexafluorophosphate (1.97 g, 5.19 mmol, 2.20 eq) followed by (5-aminopentyl)triphenylphosphonium bromide hydrobromide (2.40 g, 4.71 mmol, 2.00 eq) and anhydrous *N,N*-diisopropylethylamine (2.47 mL, 14.2 mmol, 6.00 eq). The reaction mixture was allowed to stir at room temperature under argon for 48 h then partitioned between water and chloroform. The layers were separated, and the aqueous layer was extracted with chloroform. The combined organic layers were washed consecutively with 5% aqueous lithium chloride, 1 M aqueous hydrochloric acid, saturated aqueous sodium bicarbonate and brine then dried over anhydrous magnesium sulfate and concentrated under reduced pressure. The resulting residue was dissolved in a minimum amount of chloroform and the crude material triturated from hexane. Column chromatography [SiO_2_, dichloromethane: methanol (100:0–80:20)] gave (Boc-CysTPP)_2_ with mixed counterions as an off-white film. Counterion exchange to give only chloride counterions gave (Boc-CysTPP)_2_•2Cl as a white hygroscopic amorphous solid (1.69 g, 61%). δ_H_ (500 MHz, DMSO-*d*_*6*_, 100°C): 1.38 [18H, s, 2 × (CH_3_)_3_CO], 1.46–1.54 (8H, m, 2 × CH_2_-3 and CH_2_-4), 1.57–1.66 (4H, m, 2 × CH_2_-2), 2.92–3.07 (6H, m, 2 × SC*H*^A^H^B^ and 2 × CH_2_N), 3.15 (2H, dd, 13.3 and 4.6 Hz, 2 × SCH^A^*H*^*B*^), 3.48–3.54 (4H, m, 2 × CH_2_P), 4.08–4.23 (2H, m, 2 × CHN), 6.54–6.60 (2H, m, 2 × N*H*Boc), 7.73–7.93 (32H, m, 2 × PPh_3_, 2 × N*H*CH_2_); δ_C_ (126 MHz, DMSO-*d*_*6*_): 20.3 (d, *J* = 48.8 Hz, CH_2_P), 21.5 (d, *J* = 4.1 Hz, CH_2_-2), 27.0 (d, *J* = 17.7 Hz, CH_2_-3), 28.1 (CH_2_-4), 28.2 (CH_3_), 38.1 (CH_2_N), 40.9 (CH_2_S), 53.8 (CHN), 78.3 (C of ^t^Bu), 118.6 (d, *J* = 85.6 Hz, C-P), 130.3 (d, *J* = 12.4 Hz, CH *meta* to P), 133.6 (d, *J* = 10.1 Hz, CH *ortho* to P), 134.9 (d, *J* = 2.6 Hz, CH *para* to P), 155.2 (carbamate C=O), 170.2 (amide C=O); δ_P_ (67 MHz, DMSO-*d*_*6*_): 24.4; HRMS (ESI^+^, *m/z*): found [M]^2+^ 550.2431. C_62_H_78_N_4_O_6_P_2_S_2_^2+^ requires 550.2414. IR (cm^−1^): 1435 (P-Ph), 1662 (C=O), 1705 (C=O), 2904 (CH), 2972 (CH), 3346 (NH), 3442 (NH); [α]_D_^18^ = −105.2° (*c* 1, methanol). Data assignment assisted by COSY, DEPT135 and HSQC 2D NMR spectra.

##### Disulfide of [5-(2(R)-amino-3-mercaptopropanoylamino)pentyl]triphenylphosphonium chloride, bis-hydrochloride salt, (CysTPP)_2_•2Cl•2HCl

To a solution of the disulfide of {5-[3-mercapto-2(*R*)-(*tert*-butoxycarbonylamino)propanoylamino]pentyl} triphenylphosphonium chloride (1.32 g, 1.12 mmol, 1.00 eq) in methanol (10.1 mL) at 0°C was added concentrated hydrochloric acid (4.90 mL) dropwise. The reaction mixture was allowed to stir at room temperature for 16 h then concentrated under reduced pressure. Trituration from diethyl ether gave (CysTPP)_2_•2Cl•2HCl as a white hygroscopic amorphous solid (1.17 g, 99%). δ_H_ (400 MHz, DMSO-*d*_*6*_): 1.41–1.57 (12H, m, 2 × CH_2_-2, CH_2_-3 and CH_2_-4), 3.02–3.10 (4H, m, 2 × CH_2_N), 3.26 (2H, dd, 13.9 and 7.3 Hz, 2 × SC*H*^A^H^B^), 3.41 (2H, dd, 13.9 and 5.7 Hz, 2 × SCH^A^*H*^*B*^, partly obscured by H_2_O), 3.59–3.57 (4H, m, 2 × CH_2_P), 4.09–4.14 (2H, m, 2 × CHN), 7.73–7.92 (30H, m, 2 × PPh_3_), 8.72 (6H, s, 2 × NH_3_), 9.17 (2H, t, *J* = 5.8 Hz, 2 × CONH); δ_C_ (101 MHz, DMSO-*d*_*6*_): 20.7 (d, *J* = 49.7 Hz, CH_2_P), 21.9 (d, *J* = 3.9 Hz, CH_2_-2), 27.4 (d, *J* = 16.9 Hz, CH_2_-3), 28.2 (CH_2_-4), 38.7 (CH_2_N), 39.3 (CH_2_S), 51.8 (CHN), 119.1 (d, *J* = 85.5 Hz, C-P), 130.7 (d, *J* = 12.4 Hz, CH *meta* to P), 134.1 (d, *J* = 10.1 Hz, CH *ortho* to P), 135.3 (d, *J* = 1.6 Hz, CH *para* to P), 166.9 (C=O); δ_P_ (67 MHz, DMSO-*d*_*6*_): 24.6; HRMS (ESI^+^, m/z): found [M]^2+^ 450.1894. C_52_H_62_N_4_O_2_P_2_S_2_^2+^ requires 450.1889. IR (cm^−1^): 1436 (P-Ph), 1672 (C=O), 2859 (CH), 2924 (CH), 3202 (NH). [α]_D_^21^ = −21.5° (*c* 1.3, DMSO). Data assignment assisted by COSY, DEPT135 and HSQC 2D NMR.

#### Mitochondrial isolation

Crude rat liver mitochondria were isolated by differential centrifugation, with all steps being performed at 4°C using pre-cooled equipment. Rats were culled by stunning followed by cervical dislocation. Tissues were excized and immediately stored in ice-cold STE buffer (250 mM sucrose, 10 mM Tris-HCl, 1 mM EGTA, pH 7.4 at 4°C). The liver was cut into pieces before rinsing thoroughly in ice-cold STE. The tissues pieces were chopped finely with a razor blade, residual blood and connective tissue were removed, before rinsing again with STE. Tissue pieces were homogenized in a glass tube with 5 strokes of a loose-fitting Potter-Elvehjem PTFE pestle followed by 5 strokes with a tight-fitting Potter-Elvehjem PTFE pestle. The homogenate was centrifuged (1000 × *g*, 3 min, 4°C). Mitochondria were pelleted from the supernatant by centrifugation (10,000 × *g*, 10 min, 4°C). The pellet was resuspended in STE and centrifuged again (10,000 × *g*, 10 min, 4°C). Mitochondria were resuspended in STE buffer, placed on ice and used immediately. The protein concentration was subsequently determined using a BCA assay kit with BSA as standard.

#### Sample preparation for the CysTPP assay

On dry ice ∼25 mg of frozen tissue was weighed into a 2 mL tube containing 2.8 mm ceramic beads (Omni). These tubes containing tissue were stored on dry ice before addition of 10 μL of 80% (v/v) methanol/mg of tissue. After a brief period on ice to prevent tubes fracturing, the tissue was disrupted using a Precellys 24 tissue homogenizer (Bertin Instruments, France) on a 6500 setting for 15s and then immediately placed back on ice for 5 min. Samples were then re-homogenized (6,500 rpm, 15 s) and again placed on ice. Each sample was vortexed just prior to removal of 100 μL to a fresh 1.5 mL eppendorf tube. This was kept on ice before centrifugation at 16000 × g for 2 min to pellet protein. 60 μL of the clear supernatant was transferred to a fresh 1.5 mL eppendorf tube and placed on dry ice. Any remaining liquid was removed and discarded before the pellet was resuspended in 20 μL of water to release any remaining acyl-CoA trapped in the pellet. After resuspension of the pellet, 80 μL of methanol was added and the tube centrifuged at 16000 × g for 2 min to repellet protein. 60 μL of this clear supernatant was transferred to a fresh 1.5 mL eppendorf tube on dry ice. The two 60 μL extracts from each sample were then processed separately.

#### CysTPP assay

(CysTPP)_2_ was prepared as a 50 mM stock solution in DMSO. Acyl-CoA standards were stored at −80°C in 80% (v/v) methanol. Fresh TCEP (100 mM; pH 7.8, NaOH) was prepared by adding 170 μL of water to 5.6 mg of TCEP-HCl before adding sequentially 8 μL of 5 M NaOH, 20 μL of 1 M NH_4_HCO_3_ and finally 2 μL of 5 M NaOH. Sufficient CysTPP master mix (2 mM (CysTPP)_2_, 20 mM TCEP and 200 mM NH_4_HCO_3_ in 20% (v/v) DMSO) was prepared and left on ice for ∼5 min to allow TCEP to reduce (CysTPP)_2_ to CysTPP. CysTPP master mix (20 μL) was added to each 60 μL sample or standard which was then vortexed, briefly centrifuged and incubated in a shaking water bath at 37°C for 3 h. After addition of 20 μL 25 mM DTT in 80% (v/v) methanol the samples were vortexed, briefly centrifuged and incubated at 37°C for 10 min to remove acyl groups from the thiol of CysTPP. After addition of 100 μL of 100 mM IAM, 100 mM NH_4_HCO_3_ in 50% (v/v) DMSO the samples were vortexed, briefly centrifuged and incubated at 37°C for 30 min. The reaction was quenched with 300 μL of 93% (v/v) DMSO containing 0.33% (v/v) formic acid (FA) to give an acid stabilized sample in 80% (v/v) organic solvent (∼13% methanol, ∼67% DMSO). Importantly, ACN is not compatible with early steps in the CysTPP assay as it reacts with CysTPP to generate a false acetyl-CysTPP-CAM signal.

Each sample was compared to 22 acyl-CoA standards (0-5 μM of each combined in 60 μL of 80% (v/v) methanol) reacted with CysTPP as above to generate a daily five-point standard curve of their corresponding *N-*acyl-CysTPP-CAMs.

#### LC-MS/MS of acyl-CysTPP-CAMs

LC-MS/MS analyses of acyl-CysTPP-CAMs was performed using a Xevo TQ-S triple quadrupole mass spectrometer (Waters, UK). Samples in 13% methanol/67% DMSO 0.2% formic acid were analyzed as batches beginning immediately after sample preparation. Samples were kept in glass MS vials at 8°C prior to injection of 2 μL each sample using an autosampler. All acyl-Co-As with standards were analyzed within 7 h of sample preparation. Separations were performed on a I-Class ACQUITY UPLC BEH C18 column (1 × 50 mm, 130 Å, 1.7 μm; Waters, UK) with a UPLC filter (0.2 μm; Waters, UK) at 30°C using a ACQUITY UPLC I-Class system (Waters, UK). The mobile phases were MS solvent A (5% ACN, 0.1% FA) and B (90% ACN, 0.1% FA) at a flow rate of 0.2 mL/min with the following gradient: 0–1.5 min, 5% B; 1.5–2 min, 5-15% B; 2-5 min, 15% B; 5-8 min, 15-100% B; 8-9 min, 100% B. The eluate was analyzed by MS for the complete 5 min UPLC gradient. CysTPP products were detected by MRM with electrospray ionization in positive ion mode using a source spray voltage of 3.1 kV and an ion source temperature of 200°C. Cone voltages, collision energies and MS/MS transitions for individual acyl-CysTPP-CAMs are shown in Table S1. Nitrogen and argon were used as the curtain and the collision gases, respectively. The peak area of each acyl-CysTPP-CAM in each sample and standard was quantified using the MassLynx 4.1 software after automatic peak selection were manually checked and curated where necessary. After subtraction of the signal from two blanks, each containing 80% (v/v) methanol reacted with CysTPP in the same way as standards and samples, the concentration of each acyl-CysTPP-CAM was calculated by comparing the signal in each sample to the relevant standard curve (Tables S1 and S2).

#### LC-MS/MS of acylated GDH

GDH (250 μg mL^−1^) in 100 mM HEPES (pH 7.8, NaOH) was incubated alone or with 2 mM of either octanoyl-CoA or palmitoyl-CoA for 6 h at 37°C. Subsequently each 20 μL sample was incubated with 5 μL of 25 mM DTT, 5% SDS for 15 min at 37°C before thiols were carbamidomethylated with 5 μL of 200 mM IAM for 30 min at 37°C. Protein in each sample was precipitated with 20 volumes of methanol and the pellet trypsinized overnight in 100 μL 25 mM NH_4_HCO_3_/10% ACN. Tryptic peptides were injected in either 10% or 45% ACN containing 0.1% TFA. Data was acquired on an Orbitrap QExactive Plus coupled to an RSLC3000 nanoUPLC via an EASY spray source (Thermo Fisher Scientific). Peptides were fractionated using a 75 μm × 50 cm PepMap RSLC C18 column with mobile phases A (0.1% formic acid) and B (80% ACN, 0.1% formic acid). Samples were subjected to a gradient rising from 3 to 10% B by 7 mins, to 40% B by 52 mins and to 95% B by 55 min. Data was acquired using a DDA strategy with MS data acquired between 400-1500 *m/z* at 70,000 fwhm resolution.

Raw files were processed using PEAKS Studio (version 10.6 build 20201221, Bioinformatics Solutions Inc.) with the following parameters: Enzyme: Trypsin (specific), Bos Taurus database (UniProt reference proteome downloaded 05 Aug 2019 containing 23861 proteins) with additional contaminant database (containing 246 common contaminants). Fixed modification at PEAKS DB stage: carbamidomethylation (Cys). Variable modifications at PEAKS DB stage: oxidation (Met), acetylation (Lys), octanoylation (Lys; +126.1045) and palmitoylation (Lys; +238.2297).

### Quantification and statistical analysis

Statistical analysis methods used in this study are indicated in the figure legends. Unless otherwise indicated statistical significance was calculated using Prism version 9 for Mac (GraphPad) using a one-way ANOVA followed by a Tukey or Dunnet’s multiple comparison test as indicated. p < 0.05 was considered to indicate statistical significance throughout the study. For animal experiments, efforts were made to achieve this study’s scientific goals with the minimum number of animals.

## Data Availability

Raw and processed NMR spectra for (CysTPP)_2_ is available at http://dx.doi.org/10.5525/gla.researchdata.1214. All other original source data is available from AMJ (aj@mrc-mbu.cam.ac.uk) or MPM upon reasonable request. This paper does not report original code.

## Supplemental information

Document S1. Figures S1–S6

Table S1. Mitochondrial acyl-CoA concentrations, related to Figure 4

Table S2. Whole-tissue acyl-CoA concentrations, related to Figure 5

Table S3. Glutamate dehydrogenase is acylated by medium- and long-chain acyl-CoAs, related to Figure 6

Document S2. Article plus supplemental information

## References

[bib1] Aliev M.K., Dos Santos P., Hoerter J.A., Soboll S., Tikhonov A.N., Saks V.A. (2002). Water content and its intracellular distribution in intact and saline perfused rat hearts revisited. Cardiovasc. Res..

[bib2] Baeza J., Smallegan M.J., Denu J.M. (2016). Mechanisms and dynamics of protein acetylation in mitochondria. Trends Biochem. Sci..

[bib3] Bizzozero O.A., Bixler H.A., Pastuszyn A. (2001). Structural determinants influencing the reaction of cysteine-containing peptides with palmitoyl-coenzyme A and other thioesters. Biochim. Biophys. Acta.

[bib4] Blachnio-Zabielska A.U., Koutsari C., Jensen M.D. (2011). Measuring long-chain acyl-coenzyme A concentrations and enrichment using liquid chromatography/tandem mass spectrometry with selected reaction monitoring. Rapid Commun. Mass Spectrom..

[bib5] Cohen T.J., Friedmann D., Hwang A.W., Marmorstein R., Lee V.M. (2013). The microtubule-associated tau protein has intrinsic acetyltransferase activity. Nat. Struct. Mol. Biol..

[bib6] Dawson P.E., Muir T.W., Clark-Lewis I., Kent S.B. (1994). Synthesis of proteins by native chemical ligation. Science.

[bib7] Deutsch J., Grange E., Rapoport S.I., Purdon A.D. (1994). Isolation and quantitation of long-chain acyl-coenzyme a esters in brain tissue by solid-phase extraction. Anal. Biochem..

[bib8] Fulmer G.R., Miller A.J.M., Sherden N.H., Gottlieb H.E., Nudelman A., Stoltz B.M., Bercaw J.E., Goldberg K.I. (2010). NMR chemical shifts of trace impurities: common laboratory solvents, organics, and gases in deuterated solvents relevant to the organometallic chemist. Organometallics.

[bib9] Gray G.A. (1973). Carbon-13 nuclear magnetic resonance of organophosphorus compounds. VIII. Triphenylphosphoranes and triphenylphosphonium salts. J. Am. Chem. Soc..

[bib10] Halestrap A.P. (1989). The regulation of the matrix volume of mammalian mitochondria in vivo and in vitro and its role in the control of mitochondrial metabolism. Biochim. Biophys. Acta..

[bib11] Hansen B.K., Gupta R., Baldus L., Lyon D., Narita T., Lammers M., Choudhary C., Weinert B.T. (2019). Analysis of human acetylation stoichiometry defines mechanistic constraints on protein regulation. Nat. Commun..

[bib12] James A.M., Hoogewijs K., Logan A., Hall A.R., Ding S., Fearnley I.M., Murphy M.P. (2017). Non-enzymatic N-acetylation of lysine residues by acetylCoA often occurs via a proximal S-acetylated thiol intermediate sensitive to glyoxalase II. Cell. Rep..

[bib13] James A.M., Smith A.C., Ding S., Houghton J.W., Robinson A.J., Antrobus R., Fearnley I.M., Murphy M.P. (2020). Nucleotide-binding sites can enhance N-acylation of nearby protein lysine residues. Sci. Rep..

[bib14] James A.M., Smith A.C., Smith C.L., Robinson A.J., Murphy M.P. (2018). Proximal cysteines that enhance lysine N-acetylation of cytosolic proteins in mice are less conserved in longer-living species. Cell Rep..

[bib15] James A.M., Smith C.L., Smith A.C., Robinson A.J., Hoogewijs K., Murphy M.P. (2018). The causes and consequences of nonenzymatic protein acylation. Trends Biochem. Sci..

[bib40] Kanfi Y., Naiman S., Amir G., Peshti V., Zinman G., Nahum L., Bar-Joseph Z., Cohen H.Y. (2012). The sirtuin SIRT6 regulates lifespan in male mice. Nature.

[bib16] Kirby A.J. (1980). Effective molarities for intramolecular reactions. Adv. Phys. Organ. Chem..

[bib17] Krahenbuhl S., Krahenbuhl-Glauser S., Stucki J., Gehr P., Reichen J. (1992). Stereological and functional analysis of liver mitochondria from rats with secondary biliary cirrhosis: impaired mitochondrial metabolism and increased mitochondrial content per hepatocyte. Hepatology.

[bib18] Liu X., Sadhukhan S., Sun S., Wagner G.R., Hirschey M.D., Qi L., Lin H., Locasale J.W. (2015). High-resolution metabolomics with acyl-CoA profiling reveals widespread remodeling in response to diet. Mol. Cell. Proteomics.

[bib19] Logan A., Cocheme H.M., Li Pun P.B., Apostolova N., Smith R.A., Larsen L., Larsen D.S., James A.M., Fearnley I.M., Rogatti S. (2014). Using exomarkers to assess mitochondrial reactive species in vivo. Biochim. Biophys. Acta.

[bib20] McDonnell E., Peterson B.S., Bomze H.M., Hirschey M.D. (2015). Sirt3 regulates progression and development of diseases of aging. Trends Endocrinol. Metab..

[bib21] Palladino A.A., Chen J., Kallish S., Stanley C.A., Bennett M.J. (2012). Measurement of tissue acyl-CoAs using flow-injection tandem mass spectrometry: acyl-CoA profiles in short-chain fatty acid oxidation defects. Mol. Genet. Metab..

[bib22] Pan H., Finkel T. (2017). Key proteins and pathways that regulate lifespan. J. Biol. Chem..

[bib23] Peng C., Lu Z., Xie Z., Cheng Z., Chen Y., Tan M., Luo H., Zhang Y., He W., Yang K. (2011). The first identification of lysine malonylation substrates and its regulatory enzyme. Mol. Cell. Proteomics.

[bib24] Pietrocola F., Galluzzi L., Bravo-San Pedro J.M., Madeo F., Kroemer G. (2015). Acetyl coenzyme A: a central metabolite and second messenger. Cell Metab..

[bib25] Prus G., Hoegl A., Weinert B.T., Choudhary C. (2019). Analysis and interpretation of protein post-translational modification site stoichiometry. Trends Biochem. Sci..

[bib26] Rardin M.J., Newman J.C., Held J.M., Cusack M.P., Sorensen D.J., Li B., Schilling B., Mooney S.D., Kahn C.R., Verdin E. (2013). Label-free quantitative proteomics of the lysine acetylome in mitochondria identifies substrates of Sirt3 in metabolic pathways. Proc. Natl. Acad. Sci. U S A.

[bib27] Satoh A., Brace C.S., Rensing N., Cliften P., Wozniak D.F., Herzog E.D., Yamada K.A., Imai S. (2013). Sirt1 extends life span and delays aging in mice through the regulation of Nk2 homeobox 1 in the DMH and LH. Cell Metab..

[bib28] Tabula Muris C. (2020). A single-cell transcriptomic atlas characterizes ageing tissues in the mouse. Nature.

[bib29] Tan M., Peng C., Anderson K.A., Chhoy P., Xie Z., Dai L., Park J., Chen Y., Huang H., Zhang Y. (2014). Lysine glutarylation is a protein posttranslational modification regulated by Sirt5. Cell Metab..

[bib30] Trub A.G., Hirschey M.D. (2018). Reactive acyl-CoA species modify proteins and induce carbon stress. Trends Biochem. Sci..

[bib31] Wagner G.R., Bhatt D.P., O'Connell T.M., Thompson J.W., Dubois L.G., Backos D.S., Yang H., Mitchell G.A., Ilkayeva O.R., Stevens R.D. (2017). A class of reactive acyl-CoA species reveals the non-enzymatic origins of protein acylation. Cell Metab..

[bib32] Wagner G.R., Hirschey M.D. (2014). Nonenzymatic protein acylation as a carbon stress regulated by sirtuin deacylases. Mol. Cell.

[bib33] Wagner G.R., Payne R.M. (2013). Widespread and enzyme-independent Nε-acetylation and Nε-succinylation of proteins in the chemical conditions of the mitochondrial matrix. J. Biol. Chem..

[bib34] Weinert B.T., Moustafa T., Iesmantavicius V., Zechner R., Choudhary C. (2015). Analysis of acetylation stoichiometry suggests that Sirt3 repairs nonenzymatic acetylation lesions. EMBO J..

[bib35] Weinert B.T., Satpathy S., Hansen B.K., Lyon D., Jensen L.J., Choudhary C. (2017). Accurate quantification of site-specific acetylation stoichiometry reveals the impact of sirtuin deacetylase CobB on the E. Coli acetylome. Mol. Cell. Proteomics.

[bib36] Weinert B.T., Scholz C., Wagner S.A., Iesmantavicius V., Su D., Daniel J.A., Choudhary C. (2013). Lysine succinylation is a frequently occurring modification in prokaryotes and eukaryotes and extensively overlaps with acetylation. Cell Rep..

[bib37] Woo H.K., Go E.P., Hoang L., Trauger S.A., Bowen B., Siuzdak G., Northen T.R. (2009). Phosphonium labeling for increasing metabolomic coverage of neutral lipids using electrospray ionization mass spectrometry. Rapid Commun. Mass Spectrom..

[bib38] Yu J., Loh K., Song Z.Y., Yang H.Q., Zhang Y., Lin S. (2018). Update on glycerol-3-phosphate acyltransferases: the roles in the development of insulin resistance. Nutr. Diabetes.

[bib39] Zheng Z., Li G., Wu C., Zhang M., Zhao Y., Liang G. (2017). Intracellular synthesis of D-aminoluciferin for bioluminescence generation. Chem. Commun. (Camb.).

