# Supplementary material for: Native chemical ligation approach to sensitively probe tissue acyl-CoA pools
Source: Cell Chem Biol. 2022 Jul 21;29(7):1232–1244.e5. doi: 10.1016/j.chembiol.2022.04.005 (PMC9586882; doi:10.1016/j.chembiol.2022.04.005)
Supplement: Document S1. Figures S1–S6 [file mmc1.pdf]

Cell Chemical Biology, Volume *29*

Supplemental information

Native chemical ligation approach to sensitively probe tissue acyl-CoA pools

Andrew M. James, Abigail A.I. Norman, Jack W. Houghton, Hiran A. Prag, Angela Logan, Robin Antrobus, Richard C. Hartley, and Michael P. Murphy

Figure S1 related to Figure 1

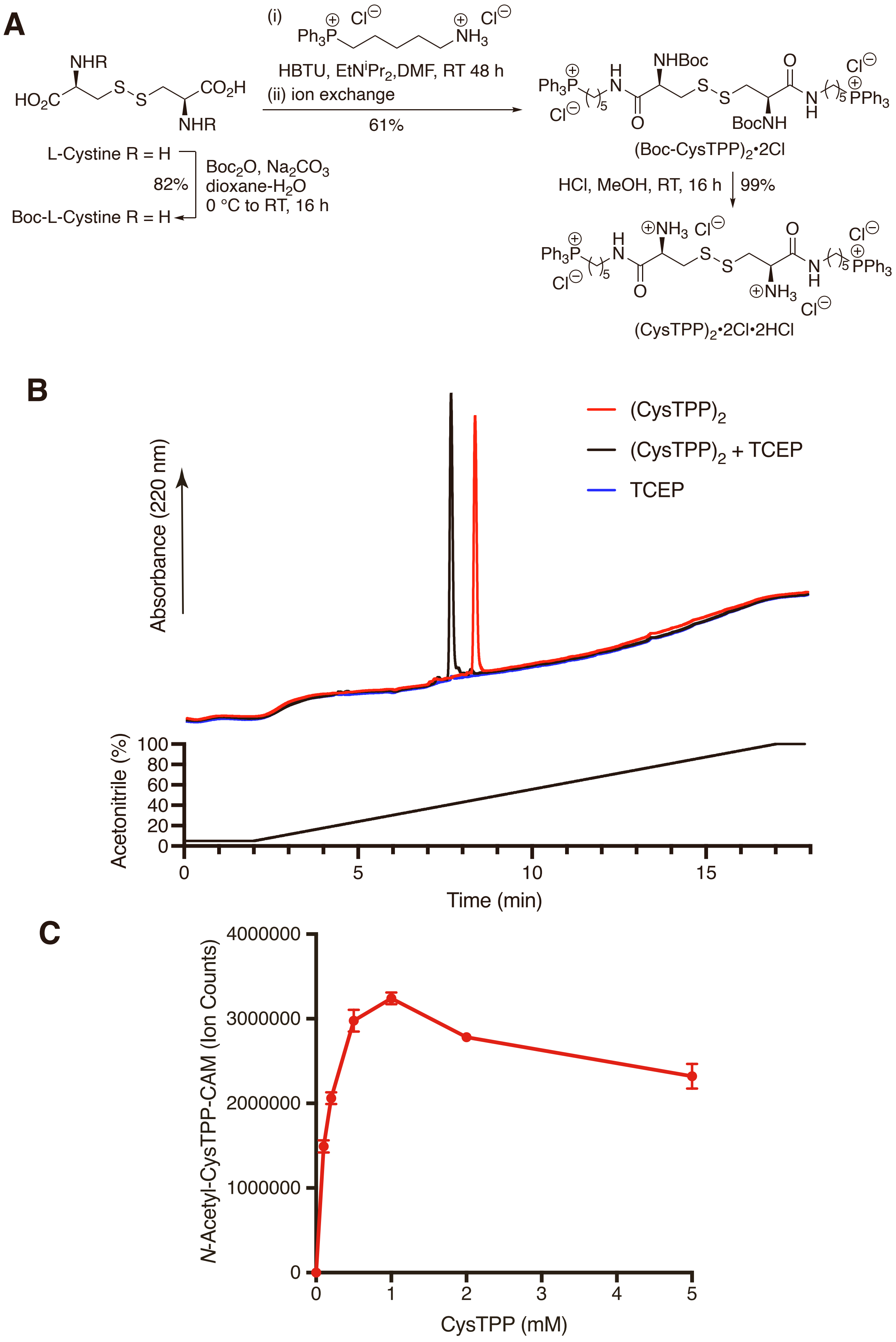

Figure S1. Synthesis of pure stable $(CysTPP)_2$ and its reduction by TCEP

A, synthesis of $(CysTPP)_2$. $(CysTPP)_2$ was synthesized in three steps from L-cystine. Boc protection of the amino groups to give Boc-L-cystine was followed by coupling with the (5-aminopentyl)triphenylphosphonium cation to give the $(Boc\text{-}CysTPP)_2$•2Cl after ion exchange. Deprotection then gave $(CysTPP)_2$, with both amino groups protonated and four chlorides as counterions, $(CysTPP)_2$•2Cl•2HCl. B, HPLC of $(CysTPP)_2$ (20 nmol; red). $(CysTPP)_2$ was reduced to CysTPP by a 20-fold excess of TCEP (black). TCEP alone (blue). C, optimization of the assay CysTPP concentration. Incubation of 200 µM acetyl-CoA with increasing concentrations of CysTPP for 3 h at 37 ˚C. After reaction with IAM the *N*-acetyl-CysTPP-CAM that was generated was quantified by LC-MS/MS using the 550→459 *m/z* transition.

Figure S2 related to Figure 1

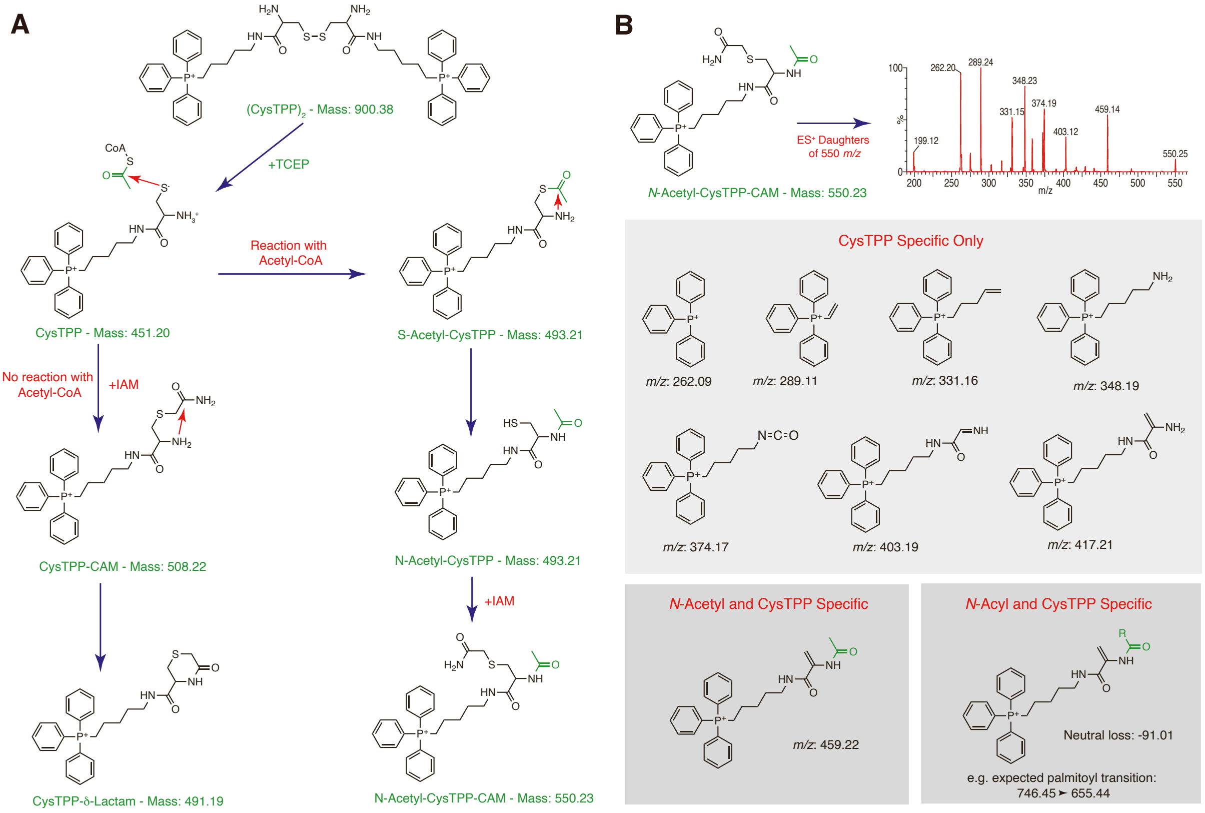

Figure S2. CysTPP Assay

A, stable $CysTPP_2$ (500 µM) is reacted with 5 mM TCEP to generate 1 mM CysTPP. Addition of acetyl-CoA results in a relatively rapid thioester exchange reaction generating CoA and an *S*-acetyl-CysTPP intermediate. The thioester carbonyl is then attacked by the proximal amine of CysTPP. The high local concentration of the amine greatly enhances what would normally be a slow *S*→*N* reaction from bulk solvent, thereby leading to the generation of *N*-acetyl-CysTPP. Any remaining *S*-acetyl groups are removed from CysTPP by the addition of 5 mM DTT and the free thiols of CysTPP and *N*-acetyl-CysTPP are blocked by the addition of 100 mM IAM. Finally, after reaction with IAM a proportion of CysTPP-CAM appears to cyclize to a δ-lactam. B, fragmentation of *N*-acetyl-CysTPP-CAM generates several TPP-containing daughter ions. However, the majority of these ions are not specific for *N*-acetyl-CysTPP-CAM as they are also generated during fragmentation of the non-acylated probe CysTPP-CAM and other *N*-acyl-CysTPP-CAM species. The exception is the peak at 459 *m/z* that still contains the acetyl moiety. Fragmentation of this same C-S bond within other *N*-acyl-CysTPP-CAM molecules is also favoured and results in a diagnostic -91 Da neutral loss to a fragment that has a molecular memory of the acyl species.

Figure S3 related to Figure 2

A

Name	Other Name	Numerical
Malonyl-CoA		
Succinyl-CoA		
Glutatryl-CoA		
HMG-CoA	3-Hydroxy-3-methylglutaryl-CoA	
ß-Hydroxybutyryl-CoA	3-Hydroxybutanoyl-CoA	
Isovaleryl-CoA	3-Methylbutanoyl-CoA	
Acetyl-CoA		C2:0-CoA
Propionyl-CoA	Propanoyl-CoA	C3:0-CoA
Butyryl-CoA	Butanoyl-CoA	C4:0-CoA
Crotonyl-CoA	(*E*)-But-2-enoyl-CoA	C4:1-CoA (n-1)
Caproyl-CoA	Hexanoyl-CoA	C6:0-CoA
Capryloyl-CoA	Octanoyl-CoA	C8:0-CoA
Caprinoyl-CoA	Decanoyl-CoA	C10:0-CoA
Lauroyl-CoA	Dodecanoyl-CoA	C12:0-CoA
Myristoyl-CoA	Tetradecanoyl-CoA	C14:0-CoA
Palmitoyl-CoA	Hexadecanoyl-CoA	C16:0-CoA
Palmitoleoyl-CoA	(*Z*)-Hexadec-9-enoyl-CoA	C16:1-CoA (n-7)
Stearoyl-CoA	Octadecanoyl-CoA	C18:0-CoA
Oleoyl-CoA	(*Z*)-Octadec-9-enoyl-CoA	C18:1-CoA (n-9)
Linoleoyl-CoA	(9*Z*,12*Z*)-Octadeca-9,12-dienoyl-CoA	C18:2-CoA (n-6)
Arachidonyl-CoA	(5*Z*,8*Z*,11*Z*,14*Z*)-Icosa-5,8,11,14-tetraenoyl-CoA	C20:4-CoA (n-6)
Cervonoyl-CoA	(4*Z*,7*Z*,10*Z*,13*Z*,16*Z*,19*Z*)-Docosahexa-4,7,10,13,16,19-enoyl-CoA	C22:6-CoA (n-3)

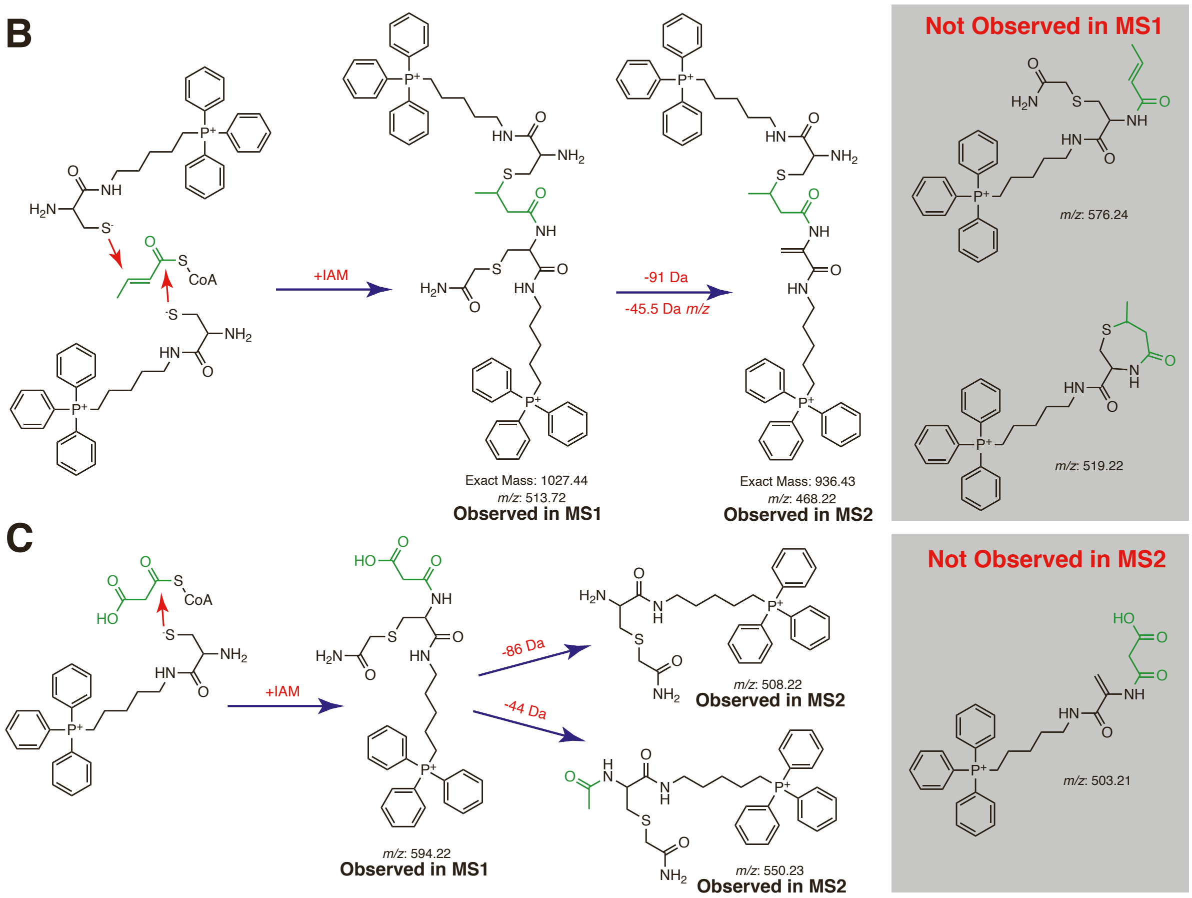

Figure S3. Fragmentation patterns are different for *trans*-2 acyl- and some carboxyacyl-CoAs

A, nomenclature of acyl-CoA standards used in this study. B, *trans*-2 acyl-CoAs can crosslink two CysTPP molecules. The reaction of the α,ß-unsaturated carbonyl of crotonyl-CoA with CysTPP leads to an alkykation as well as an *S*→*S*→*N* acyl-transfer reaction. Consequently, the diagnostic -91 Da neutral loss still occurs but presents as a -45.5 *m/z* shift because the MS1 product is a dication. No *N*-crotonyl-CysTPP-CAM or cyclic products were observed. C, reaction of malonyl-CoA with CysTPP leads to the expected malonyl-CysTPP-CAM product at 594 *m/z*, but demalonylation (-86 *m/z*) is the preferred fragmentation. A similar fragmentation to a species at 508*m/z* occurs with succinyl-CoA and HMG-CoA. Additionally, decarboxylation (-44 *m/z*) leads to small quantities of acetyl-CysTPP-CAM which are indistinguishable from the acetyl-CysTPP-CAM that arises from acetyl-CoA.

Figure S4 related to Figure 3

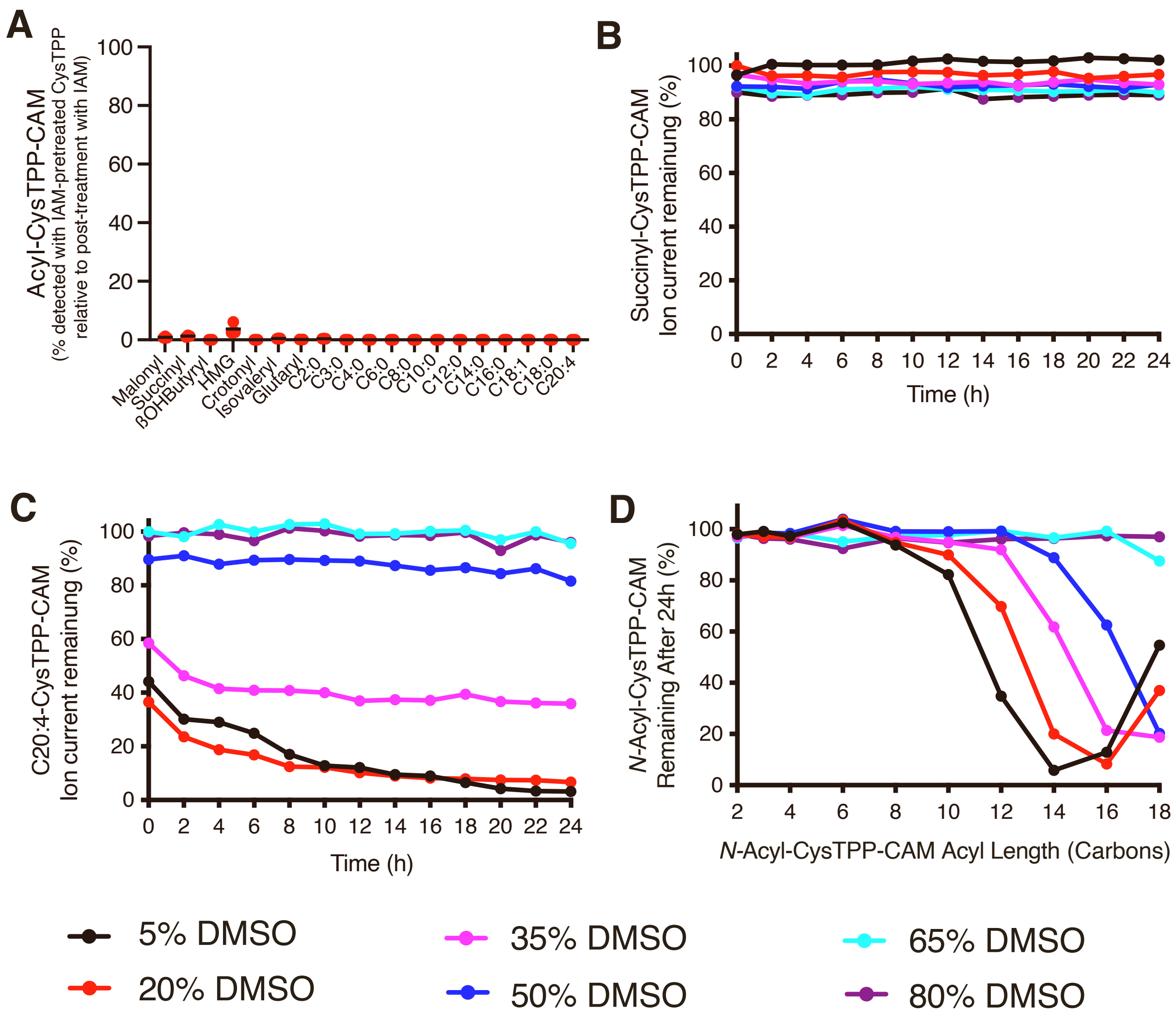

Figure S4. Dependence of acyl-CysTPP-CAM formation on the thiol of CysTPP and the stability of these products in solution

A, *N*-acylation of CysTPP by all acyl-CoAs is almost completely thiol-dependent. CysTPP was either treated with 50 µM IAM for 30 min at 37˚C after incubation for 3 h with acyl-CoA or treated with an equivalent amount of IAM for 30 min at 37 ˚C prior to incubation for 3 h with acyl-CoAs. Data is expressed as the percentage of acyl-CysTPP-CAM detected with IAM-pretreated CysTPP versus that detected with IAM posttreatment ± SEM (n=3). B, C and D, a mixture of 22 acyl-CoAs (5 µM of each) was reacted with CysTPP and derivatized with IAM. Acyl-CysTPP-CAMs were solubilized in varying concentrations of DMSO and their concentration was measured initially within 1 h and again every 2 h until 24 h at 8 ˚C. Data is the percentage of ion current remaining after 24 h relative to the maximum ion current

observed at any of the DMSO concentrations initially (~0 h). B, stability of succinyl-CysTPP-CAM in varying concentrations of DMSO over 24 h. C, stability of arachidonyl-CysTPP-CAM (C20:4) in varying concentrations of DMSO over 24 h. D, stability of saturated acyl-CysTPP-CAMs in varying concentrations of DMSO over 24 h is a function of their acyl chain length.

Figure S5 related to Figure 4

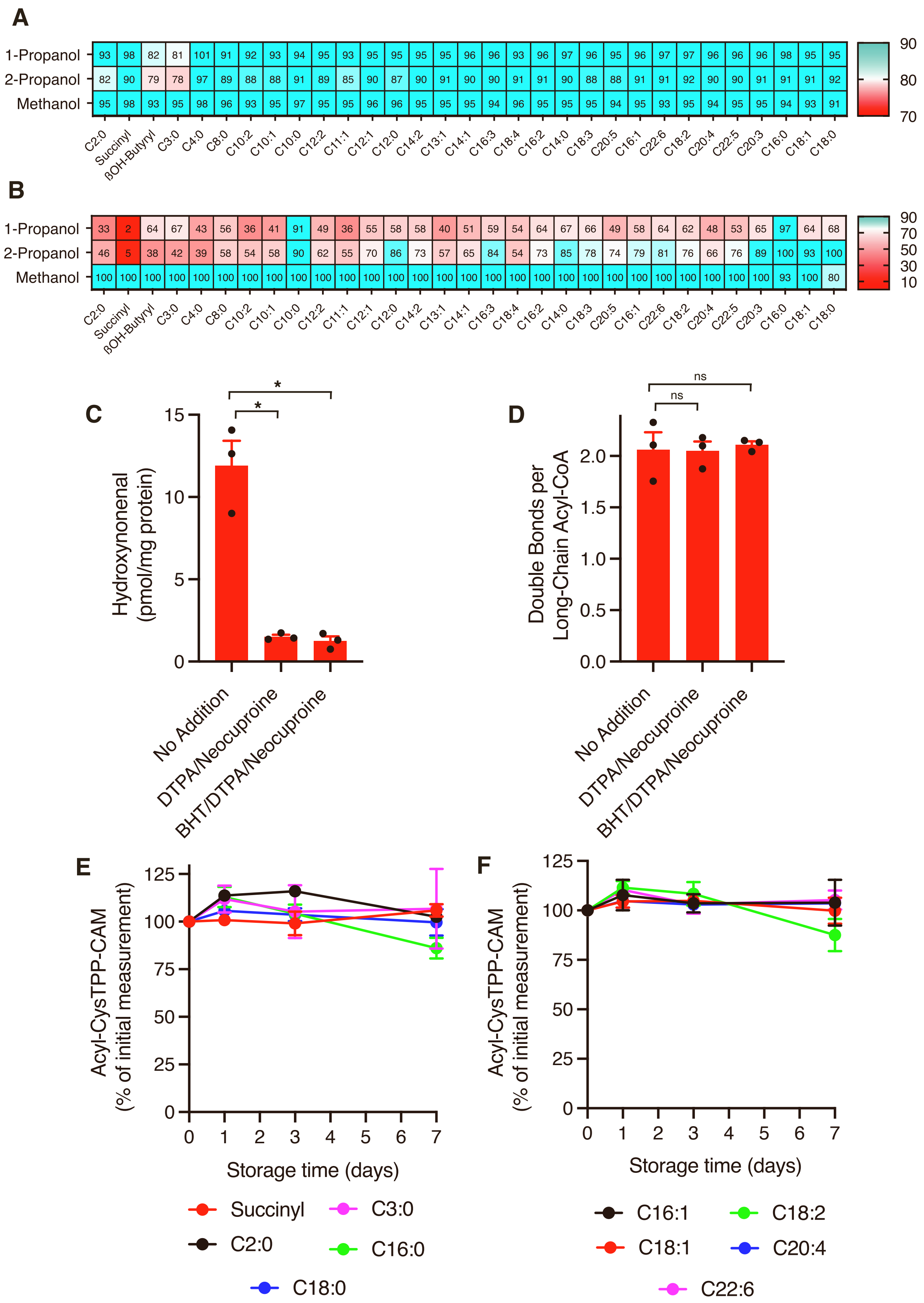

Figure S5. Optimization of solvent extraction from mitochondria and the stability of acyl-CysTPP-CAM products within extracted samples

A, two extractions with 80% (v/v) methanol extract >90% of the 31 most abundant acyl-CysTPP-CAM species from isolated mitochondrial fractions from rat liver. Values are for acyl-CysTPP-CAM recovered with two extractions with 80% (v/v) solvent relative to that from three extractions with 80% (v/v) of the same solvent. B, 80% (v/v) methanol has superior recovery of most acyl-CysTPP-CAM species. Values are for acyl-CysTPP-CAMs recovered with two extractions with 80% (v/v) solvent relative to two extractions with 80% (v/v) of the best solvent for each species. C, chelators and chain-breaking antioxidants limit the formation of 4-hydroxynonenal (HNE) during the CysTPP assay. Incubation of pure HNE with CysTPP forms a product with a 607 -> 348 *m/z* transition at a retention time of 6.1 min. Inclusion of diethylenetriaminepentaacetic acid (DTPA; 100 µM), neocuproine (100 µM) and butylated hydroxytoluene (BHT; 1 mM) to a methanol extraction of isolated mitochondrial fractions from rat liver limits the formation of this HNE-specific product. HNE was quantified relative to an HNE standard curve and is the mean ± SEM (n=3). Significance was calculated using a one-way ANOVA followed by a Tukey multiple comparison test. *, $p<0.05$. D, unsaturated acyl-CoAs are not oxidized during their extraction and subsequent incubation with CysTPP. The degree of unsaturation of long-chain acyl-CoAs extracted from rat liver isolated mitochondrial fractions is unaffected by the presence of DTPA (100 µM), neocuproine (100 µM) and BHT (1 mM). Data is the mean ± SEM (n=3) of the average number of double-bonds per long-chain (C13-C22) acyl-CoA in each sample. Significance was calculated using a one-way ANOVA followed by a Tukey multiple comparison test. ns, not significant. E and F, common saturated and unsaturated acyl-CysTPP-CAM species generated from rat liver isolated mitochondrial extracts are stable at 4-8˚C. Samples were reanalysed after storage and the acyl-CoA concentration was calculated relative to equivalently stored standards. Data is expressed as a percentage of the acyl-CoA concentration in the initial analysis ± SEM (n=3).

Figure S6 related to Figure 6

A

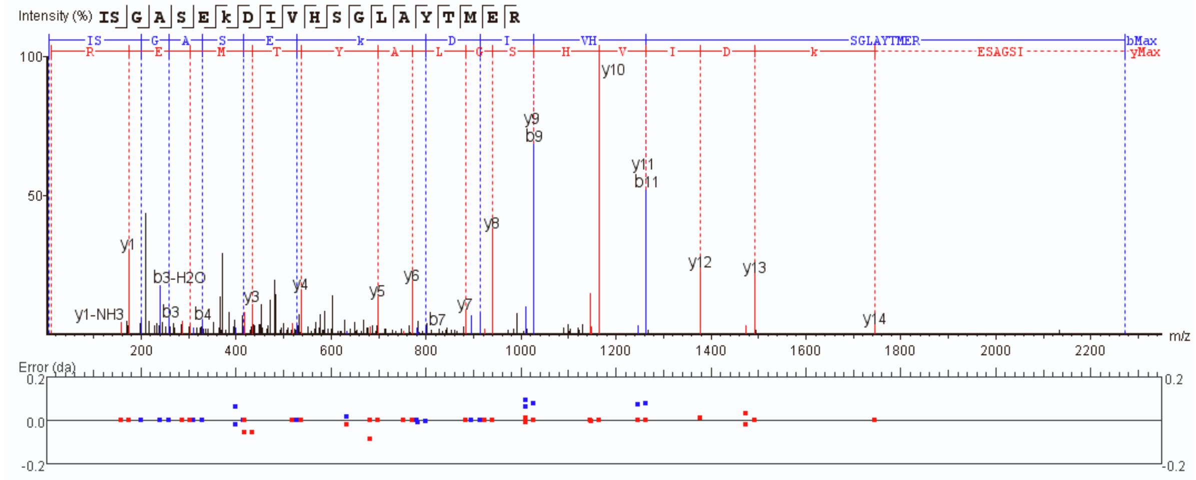

B

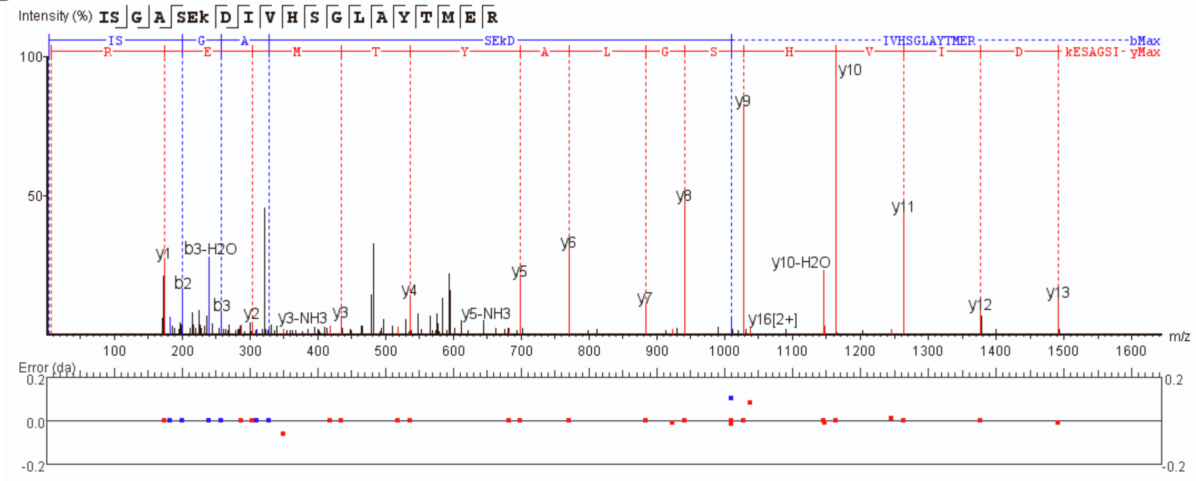

Figure S6. MS2 fragment spectra of acylated peptides

Purified GDH was treated with 2 mM octanoyl-CoA or palmitoyl-CoA for 6 h at 37 ˚C. GDH was precipitated with 90% methanol before the resulting pellet was trypsinized overnight in 10% ACN before loading in 50% ACN. A, MS2 fragmentation spectra of an octanoylated tryptic fragment containing lysine 503 (K503). B, MS2 fragmentation spectra of a palmitoylated tryptic fragment containing K503.

Table S1. Mitochondrial acyl-CoA concentrations

Table S2. Whole tissue acyl-CoA concentrations

Table S3. Glutamate dehydrogenase is acylated by medium- and long-chain acyl-CoAs
